# Spatial relationships in the urothelial and head and neck tumor microenvironment predict response to combination immune checkpoint inhibitors

**DOI:** 10.1038/s41467-024-46450-1

**Published:** 2024-03-21

**Authors:** Alberto Gil-Jimenez, Nick van Dijk, Joris L. Vos, Yoni Lubeck, Maurits L. van Montfoort, Dennis Peters, Erik Hooijberg, Annegien Broeks, Charlotte L. Zuur, Bas W. G. van Rhijn, Daniel J. Vis, Michiel S. van der Heijden, Lodewyk F. A. Wessels

**Affiliations:** 1 Department of Molecular Carcinogenesis, The Netherlands Cancer Institute, Amsterdam, the Netherlands; 2 Oncode Institute, Utrecht, the Netherlands; 3 Department of Medical Oncology, The Netherlands Cancer Institute, Amsterdam, the Netherlands; 4 Department of Head and Neck Surgery and Oncology, The Netherlands Cancer Institute, Amsterdam, The Netherlands; 5 Division of Tumor Biology & Immunology, The Netherlands Cancer Institute, Amsterdam, The Netherlands; 6 Department of Pathology, The Netherlands Cancer Institute, Amsterdam, the Netherlands; 7 Core Facility Molecular Pathology & Biobanking, Netherlands Cancer Institute, Amsterdam, Netherlands; 8 Department of Otorhinolaryngology Head and Neck Surgery, Leiden University Medical Center Leiden, The Netherlands; 9 Department of Urology, The Netherlands Cancer Institute, Amsterdam, Netherlands; 10 Department of Urology, Caritas St. Josef Medical Centre, University of Regensburg, Regensburg, Germany; 11 Faculty of Electrical Engineering, Mathematics and Computer Science, Delft University of Technology, Delft, the Netherlands

**Keywords:** Tumour biomarkers, Cancer microenvironment, Predictive markers, Bladder cancer, Oral cancer

## Abstract

Immune checkpoint inhibitors (ICI) can achieve remarkable responses in urothelial cancer (UC), which may depend on tumor microenvironment (TME) characteristics. However, the relationship between the TME, usually characterized by immune cell density, and response to ICI is unclear. Here, we quantify the TME immune cell densities and spatial relationships (SRs) of 24 baseline UC samples, obtained before pre-operative combination ICI treatment, using multiplex immunofluorescence. We describe SRs by approximating the first nearest-neighbor distance distribution with a Weibull distribution and evaluate the association between TME metrics and ipilimumab+nivolumab response. Immune cell density does not discriminate between response groups. However, the Weibull SR metrics of CD8^+^ T cells or macrophages to their closest cancer cell positively associate with response. CD8^+^ T cells close to B cells are characteristic of non-response. We validate our SR response associations in a combination ICI cohort of head and neck tumors. Our data confirm that SRs, in contrast to density metrics, are strong biomarkers of response to pre-operative combination ICIs.

## Abstract (web summary)

Spatial positioning of cells within the tumour microenvironment may have a function in the success of immune checkpoint immunotherapy (ICI). Here the authors analyse spatial relationships from immunohistochemistry samples prior to ICI therapy and show that CD8 T cell or macrophage proximity to cancer cells is associated with better responses.

## Introduction

Immune checkpoint inhibitors (ICI) block inhibitory signals between immune and neoplastic cells that can result in cancer cell killing^1^. Inhibitors targeting PD-1 and PD-L1 have shown durable responses in a subset of urothelial cancer (UC) patients^2,3^. Still, most tumors do not respond to treatment^1,4^, and ICI causes grade≥3 immune-related adverse events in 10% of UC patients^5^. Therefore, it is crucial to identify biomarkers that aid the stratification of responding patients so that alternative lines of treatment can be considered for non-responding patients and prevent unnecessary toxicities.

The surrounding of a tumor, known as the tumor microenvironment (TME), contains immune cells, normal epithelial cells, and fibroblasts that continuously interact^6^. Components of the TME indicative of pre-existing immunity have shown associations with response to ICI, such as CD8^+^ T cell infiltration and transcription factors related to T cell activity^7–11^. However, biomarkers do not behave consistently in UC trials. For instance, the baseline presence of CD8^+^ T cells correlates with ICI monotherapy response in the pre-operative (anti-PD-L1)^7^ or metastatic setting^12^. Still, in pre-operative ICI combination therapy (anti-PD(L)−1 + anti-CTLA-4), the response is independent of CD8^+^ T cell density^9,10^. The lack of robust response biomarkers highlights the need to dissect tumor-immune interactions in more detail^13^.

A technology enabling a TME characterization at single-cell resolution is multiplex immunofluorescence (mIF), which spatially profiles a tissue slide using multiple antibodies simultaneously^14,15^. MIF-derived metrics were found to predict anti-PD-1 and anti-PD-L1 response across different tumor types^16^, highlighting its ability to quantify crucial immune components that determine ICI response. Typically, mIF data are summarized as immune cell densities, informing about immune cell counts, and typically topologically assessed in different compartments, i.e., tumor and stroma^17^.

By definition, immune cell density and abundance metrics ignore the immune interactions relevant to an anti-tumor response^18^. Ignoring these interactions is suboptimal, as many immune interactions require proximity. For instance, a T cell receptor and antigen interaction require physical binding, which requires the cells involved to be in close proximity to each other. In contrast, an immunosuppressive TME will have few immune cells close to cancer cells due to their inability to infiltrate a tumor^19^. Such distance or adjacency patterns between cells at the TME can be measured through their spatial relationship (SR), allowing for a mathematical description amenable to downstream analysis^20^. Notably, associations between SRs at the TME and prognosis^18,21,22^ and response to monotherapy ICIs have been reported across different cancer types^23–25^. The SRs allow for quantitative exploration of the TME, providing a basis for improving our understanding of tumor immunology and scrutinizing new associations with ICI response. SRs in the UC’s TME are poorly understood and, to our knowledge, largely unexplored in pre-operative combination ICI treatments, and this study aims to address that.

Several analytical frameworks aim to measure the SRs of the TME, such as cell-cell interactions and tissue modules, using spatially resolved protein-derived data across multiple data types^19^. Methodologies are predominantly topologically based (graph-, networks-, and cell-counting-based methods) or distance-based^20^. Distance-based methods, such as the first-nearest-neighbor (1-NN) distribution, allow modeling proximity patterns within the TME using spatially-resolved data in a simple yet informative manner. Because distances following a 1-NN distribution are asymmetrical, approaches that estimate the 1-NN distribution mean^25^ provide inadequate data summaries. A common approach to model 1-NN distributions is through the cumulative distribution function (CDF), known as the G-function. Nevertheless, the downstream analyses require an additional summary by estimating the area under the curve (AUC) at a predefined threshold^26,27^. Currently, there is a lack of spatial methodologies that describe the distance distribution without using a threshold and that model variation between individuals.

In this study, we spatially profile cancer cells, T cells, macrophages, and B cells using mIF and present a methodology to quantify the TME SRs using 24 pre-operative baseline tumor resection UC samples from the NABUCCO trial^10^. In NABUCCO, pre-operative combination ICI with ipilimumab and nivolumab is administered in UC. We fit a Weibull distribution to the 1-NN distance distribution between pairwise cell relationships to extract a two-parameter describing the distribution. These spatial descriptions outperform immune cell densities when quantifying the differences in immune cell SRs between response groups to ICI. To demonstrate the generalizability of our findings, we confirm the baseline associations between SRs and response in an independent cohort of 25 mostly HPV-negative head and neck squamous carcinoma (HNSCC) patients receiving pre-operative ipilimumab and nivolumab treatment^28^.

## Results

### Multiplex immunofluorescence and modeling of immune cell densities and spatial relationships of urothelial and head and neck cancer samples

**Fig. 1:**
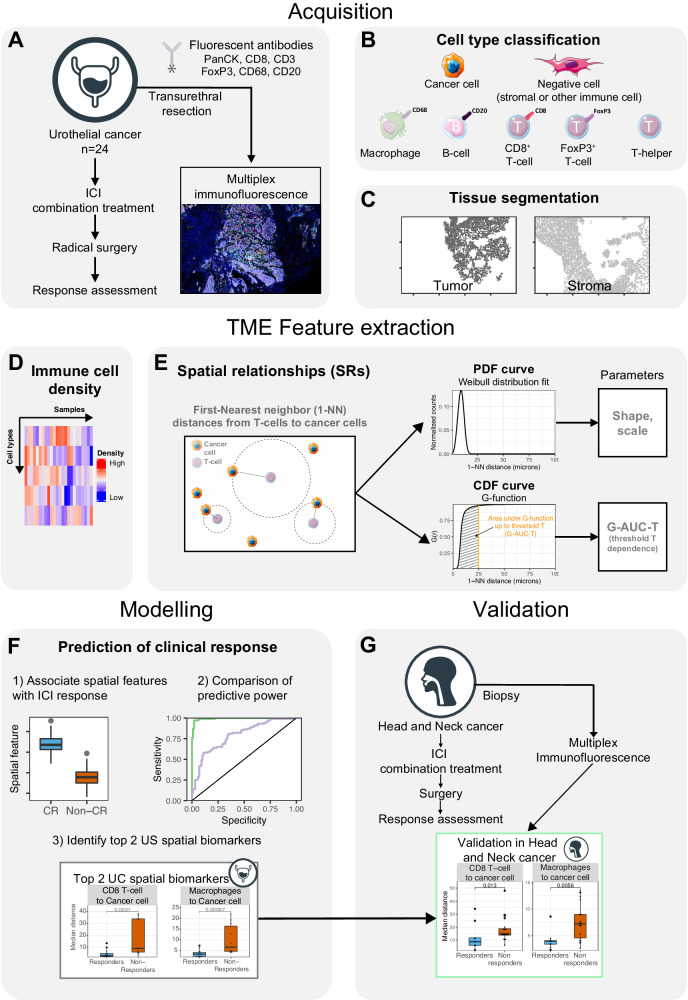
Profiling of immune cell density and spatial relationships of the urothelial cancer tumor micro-environment by multiplex immunofluorescence. **A** Biopsy samples from 24 patients from the NABUCCO trial were profiled using mIF. **B** Cell type classification by comparing antibody marker positivity. **C** Tissue segmentation into tumor and stroma regions by comparing the local densities of cancer cell marker positive and negative cells. **D** Immune cell density in the tumor and stroma compartments was calculated in each tissue compartment (tumor and stroma). **E** SRs were summarized using the 1-NN statistic studied from a reference cell type to a target cell type. The resulting 1-NN distances vector was studied using 2 approaches: modeling a Weibull distribution to the Probabilistic Density Function (PDF) (top), and using the cumulative distribution function (CDF) using the G-function. **F** Association of SR parameters with ICI response and comparison of the discriminative power between SR and density TME parameters. **G** Validation of associations between SR parameters and response identified in UC in an independent cohort of HNSCC tumors. Icons from panel A, B, F and G were adapted from bioIcons (cancerous-cell-1, lymphocytes-4, macrophage, t-lymphocyte, b-lymphocyte, fibroblast-1 licensed under CC-BY 3.0 Unported by Servier), flaticon.com (bladder icon, https://www.flaticon.com/free-icon/bladder_1453578; head neck icon, https://www.flaticon.com/free-icon/injection_4418017). TME: tumor micro-environment; SR: spatial relationship; mIF: multiplex immunofluorescence; ICI: immune checkpoint inhibitors; 1-NN: first nearest neighbor; PDF: probabilistic density function; CDF: cumulative density function; G-AUC-T: G-function evaluated at a threshold T; T: threshold; UC: urothelial cancer; HNSCC: head and neck squamous cell carcinoma. Source data are provided with this paper.

We collected multiplex immunofluorescence (mIF) data from baseline formalin-fixed, paraffin-embedded (FFPE) stage-III urothelial cancer (UC) samples (*n* = 24), of patients recruited in the NABUCCO trial (Fig. 1A, Table 1). Patients received pre-operative combination ICI, consisting of two or three cycles of ipilimumab (anti-CTLA-4) and nivolumab (anti-PD-1)^10^. We determined the position and identity of cells using mIF and identified B cells, T cells (CD8^+^ T cells, FoxP3^+^ T cells, and T-helper cells), macrophages, and cancer cells (Fig. 1B). Negative cells scored negative for all the antibodies (CD8^-^, CD3^-^, FoxP3^-^, CD20^-^, CD68^-^, PanCK^-^); this group contains all stromal cells and immune cells not covered by our antibody panel. Next, by comparing the local density of cancer and negative cells, we virtually segmented the tissue into the tumor and stroma compartments (Fig. 1C, Supplementary Fig. 1) and quantified immune cell density in both compartments (Fig. 1D, Supplementary Data 1).

**Table 1:** Clinical trial, treatment and sample characteristics for the cohorts used in this study

	NABUCCO *NCT03387761*	IMCISION *NCT03003637*
Sample size	24	25^a^
Cancer type	Urothelial cancer	Head and neck cancer
Tissue source	Bladder, *n* = 24 (100%)	Oral cavity, *n* = 24 (96%) Oropharynx, *n* = 1 (4%)
Tissue sampling	Transurethral resection (primary tumor, FFPE)	Primary tumor biopsy (FFPE)
Treatment type	Pre-operative (neoadjuvant)	Pre-operative (neoadjuvant)
Treatment dosage per cycle	1: ipilimumab 2: ipilimumab + nivolumab 3: nivolumab	1: ipilimumab + nivolumab 2: nivolumab
Tumor type	Primary, *n* = 24 (100%)	Primary, *n* = 20 (80%) Recurrence, *n* = 5 (20%)
HPV-positivity (%)	n/a	23 (92%)
Response definition	Pathological response assessment (complete pathological response or residual disease)	Pathological response assessment combined with comparison of tumor cells decrease from baseline vs. on-treatment sample
Response (%)	14 (58%)	9 (36%)
Clinical Stage	cT3-4aN0M0 *n* = 14 (58%) cT2-4aN1-3M0 *n* = 10 (42%)	cT2N0 *n* = 5 (20%) cT3-4aN0M0 *n* = 10 (40%) cT2-4aN1-3M0 *n* = 10 (40%)
Sex (%)	Male *n* = 18 (75%)	Male *n* = 20 (80%)

^a^Only samples from IMCISION Arm B (combination ICIs) have been used in this manuscript.

We quantified the pairwise SRs between all cell types in the TME using the first nearest-neighbor (1-NN) distance statistic (Supplementary Data 2). In brief, the statistic is measured between a reference cell type (cell from) and a target cell type (cell to). The distances between each reference cell type and their closest target cell type yielded a 1-NN distance vector (Fig. 1E). Then, we fitted a Weibull distribution function using a non-linear mixed effect model to summarize the 1-NN distance distribution. The model has two parameters: shape and scale, which uniquely describe the properties of the 1-NN distance distribution (Fig. 1E, top) in a threshold-independent manner. We estimated the Weibull parameters (scale and shape) for all 49 pairwise relationships between cell types for all samples using the data from the whole tissue slide. Additionally, we evaluated the threshold-dependent G-function derived from the cumulative distribution function (CDF) of the 1-NN distance distribution and broadly documented in the spatial statistics literature^29^. The G-functions were summarized by computing the area under the curve (AUC) at different thresholds T, which we refer to as G-AUC-T (which included *T* = 25 [Fig. 1E, bottom], *T* = 50 μm and *T* = 100 μm) across all pairwise cell type SRs and samples.

We then compared the spatial (Fig. 1E) and density (Fig. 1D) parameters with response to ICIs and compared their predictive power (Fig. 1F). Lastly, we validated the associations between TME parameters and response in an independent cohort of mostly HPV-negative head and neck squamous cell carcinoma (HNSCC, *n* = 25) using baseline primary tumor samples of patients recruited in the IMCISION trial that received pre-operative ipilimumab and nivolumab combination treatment (Fig. 1G).

### Exploration of spatial relationships across the urothelial cancer tumor micro-environment

**Fig. 2:**
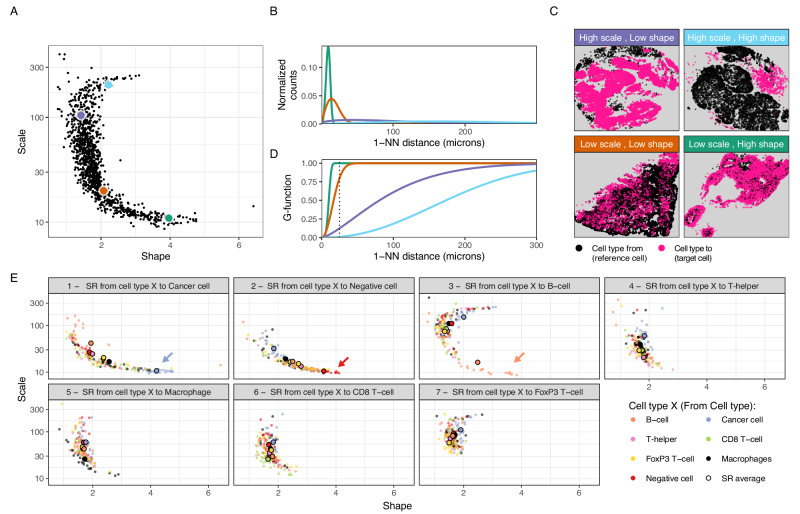
Exploration of pairwise cell type SRs in the TME using the 1-NN distance statistic. **A** Scale vs shape SR parameters fitted on the 1-NN distribution for the 24 samples and the 7 × 7 cell type combinations. Representative examples are highlighted in green, orange, purple and cyan, with their associated 1-NN distance distributions (**B**), point patterns (**C**) and G-functions (**D**). **E** Scatter plot of the scale-shape parameter space by target cell type (cell type to in the SR). For instance, the first facet represents SRs studied from any reference cell type to cancer cells. There, the coloring denotes the reference cell type (cell type from); i.e. the orange dots represent spatial relations studied from B cells to cancer cells. Cohort averages for their associated SR parameters are highlighted as big dots for each cell type-cell type combination. SR: spatial relationship; TME: Tumor microenvironment; 1-NN: First nearest neighbor. Source data are provided with this paper.

We quantified the SRs for all pairwise relationships of immune, cancer, and negative cells by estimating the Weibull parameters (shape, scale) characterizing the 1-NN distance distributions (Fig. 1E). Next, we explored the shape-scale parameter space across patients (Fig. 2A).

To illustrate what the Weibull parameters (shape, scale) represent, we use four combinations of the SR metrics that characterize distinct instances of cellular spatial distributions (Fig. 2A, colored dots). For the green dot in Fig. 2A, we observe that the 1-NN distance distribution is characterized by relatively low distances (Fig. 2B, green curve), which originate from the black cells being close to a pink cell (Fig. 2C, “Low scale, High shape”). The low scale/high shape value signals densely packed pink cells, meaning there is always a pink cell close to a black cell. Another type of SR characterized by relatively short 1-NN distances but with a higher variance in 1-NN distances, is illustrated in Fig. 2A, B (orange dot and curve). It represents an SR where one or both cell types are arranged in overlapping, densely packed clusters, such as the pink and black cells in Fig. 2C (*“*Low scale, Low shape*”*). Cases with an even larger spread in distances (Fig. 2A, B, purple dot and curve) from the black to the nearest pink cells, are described by high scale and low shape parameter values (Fig. 2C, *“*High scale, Low shape*”*). Lastly, the cyan dot (Fig. 2A) represents a 1-NN distribution shifted towards higher 1-NN distances (Fig. 2B, cyan curve), characteristic of a repulsion pattern, i.e., where both cell types are clustered in relatively large clusters (Fig. 2C, *“*High scale, High shape*”*). Therefore, increasing the scale parameter results in an increase of the distance distribution spread (i.e., the distribution width, Supplementary Fig. 2A, B), while the shape parameter is related to the distinct forms of the distribution behavior (Supplementary Fig. 2C-F, Supplementary Fig. 3).

Furthermore, the associated threshold-dependent G-function results showed corresponding differences between the four scenarios, which is expected, as the G-function is the cumulative distribution of the 1-NN distances (Fig. 2D, Eq. 4). However, in contrast to the Weibull approach, a threshold value (T) is required to generate the summary metric G-AUC-T.

**Fig. 3:**
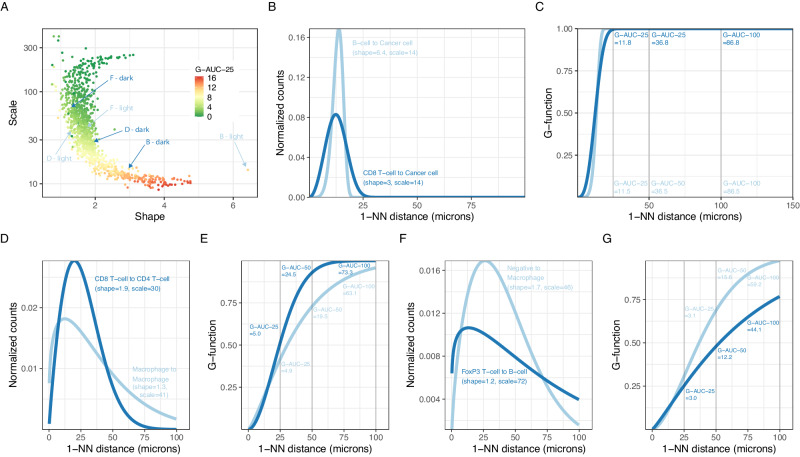
Comparison of spatial relationship parameters derived from the 1-NN distance statistic. **A** Scale/shape parameter space associated with the G-AUC-25 (coloring, G-function AUC evaluated at 25 μm). **B**, **D**, **F** 1-NN PDF distance distribution of pairs of examples with a substantial difference in the shape parameters. The plot reports each curve’s shape, scale, and SR. Each SR is annotated in (**A**). **C**, **E**, **G** Associated G-functions (CDF) of the examples shown in panel A of pairs of examples without a substantial difference in G-AUC-25. G-AUCs evaluated at different thresholds T are reported in the figure. AUC: Area under the curve; G-AUC-T: G-function AUC evaluated at a threshold T; T: threshold; 1-NN: first nearest neighbor; SR: spatial relationship. Source data are provided with this paper.

We then dissected the SRs by reference and target cell type to explore patterns of SRs across the TME (Fig. 2E). First, we investigated self-self relationships, which are relationships between cells of the same type. The self-self relationship for tumor cells falls in the “Low scale, High shape” scenario, with shorter distances between cells as tumor cells are typically densely packed in the tumor regions (Fig. 2E-1, blue dots and arrow). Similar behavior was observed for negative cells, indicating a tight packing of negative cells (Fig. 2E-2, red dots and arrow). Then, we explored the self-self relationships of immune cells. We observed B cells clustering for all patients (Fig. 2E-3, orange dots and arrow). We did not observe the same clustering behavior for self-self SRs of other immune cell types (e.g., green dots in Fig. 2E-6, which shows a behavior more akin to the *“*Low scale, Low shape*”* scenario). Subsequently, we assessed the SRs between different cell types. We observed a high variation in the Weibull parameters across samples and pairwise cell type combinations (Fig. 2E) and a dependence on the SR perspective, i.e., whether a given cell type is the cell type from or the cell type to, which can be attributed to the asymmetric property of the 1-NN statistic, as illustrated in Supplementary Fig. 4.

In short, we created a framework to quantify, interpret, and study SRs in the TME using the Weibull parameters extracted from the 1-NN distance distributions. The framework allows exploring distinct cellular organization patterns and quantifying specific SRs (e.g., T cell to B cell vs. B cell to T cell) amenable for downstream analyses aimed at furthering our understanding of the TME and its relationship to response to ICIs.

### Comparison between spatial relationship metrics derived from the first nearest neighbor distance statistic

A common approach to extracting parameters from the 1-NN distance distribution statistic is through the G-function, which represents the cumulative density function (CDF) of the 1-NN distribution and requires a particular threshold to summarize the data for downstream analyses. We compared our Weibull parameters with the G-function summary. We evaluated the G-function using its area under the curve (AUC) up to a 25-μm distance, which we defined as the “G-AUC-25”. We chose this threshold after inspecting the distance region in which the G-function showed the highest variability. Because we observed variability in G-functions across pairwise cell-type relationships, other thresholds were evaluated and denoted as “G-AUC-T”, in which T denotes the threshold in micrometers. We observed a non-linear relationship between the shape, scale and the G-AUC-25 (Fig. 3A), G-AUC-12 and G-AUC-50 (Supplementary Fig. 5A, B).

Upon summarizing the G-function, and for a given SR’s G-AUC-25 value, we observed that the associated shape and scale parameters can show substantial variation (Fig. 3A; e.g., dots colored in red mapping to a wide range of shape parameter values). Figure 3A highlights a pair of SRs with large differences between their shape or scale parameters, which can be visually confirmed by their 1-NN curves (Fig. 3B, D, F). In contrast to the Weibull parameters, the corresponding G-AUC-25 values do not differ substantially between the pair members (Fig. 3C, E, G, showing the G-function curves corresponding to the pairs in Fig. 3B, D, F, respectively), which can hinder interpretations on the associated SR. Specifically, comparing Figs. 3B, C, we observe that while the Weibull parameters are quite different (scale = 14 and shape = 6.4 for B-light, scale = 14 and shape = 3.0 for B-dark) the G-AUC-T values are quite similar (G-AUC-25 = 11.5 for B-light and G-AUC-25 = 11.8 for B-dark). While in the other examples, the differences in the G-AUC-25 values were small, we found that higher values of the summary threshold could better capture the difference between the SR pairs in Fig. 3E (Weibull density in 3D) and Fig. 3G (Weibull density in 3F).

To further illustrate differences between SRs captured by the shape or scale parameters but not by the G-AUC-25 parameter, we compared G-AUC-Ts for different pairwise cell-cell relationships (Supplementary Fig. 5C). Here, for different samples but the same SRs (e.g., Macrophages to B cell), the magnitude of the increase in the G-AUC-T value when altering the evaluation threshold T, depending on the studied pairwise cell-cell relationship, the G-function’s shape (rapidly or slowly reaching the maximum value), and the sample. In some SRs, the G-AUC’s increase was linear (e.g., Cancer cell to cancer cell) because the G-function saturated at low thresholds (Supplementary Fig. 5D). Still, in others (e.g., Macrophages to B cell), the increase was not always linear because the G-function reaches saturation at higher thresholds (Supplementary Fig. 5D). Therefore, when using a G-function statistic, such as the G-AUC-T, the SR quantification critically depends on the threshold used.

In short, our data shows that the G-function threshold introduces variance in the downstream metric G-AUC-T. Furthermore, optimizing the threshold to maximize the effect size of SR-biomarkers for treatment response creates a risk of overfitting. Therefore, different results using the same SR data can be obtained when varying the threshold, which can hinder downstream interpretation.

### Spatial relationships associated with immune checkpoint blockade response

**Fig. 4:**
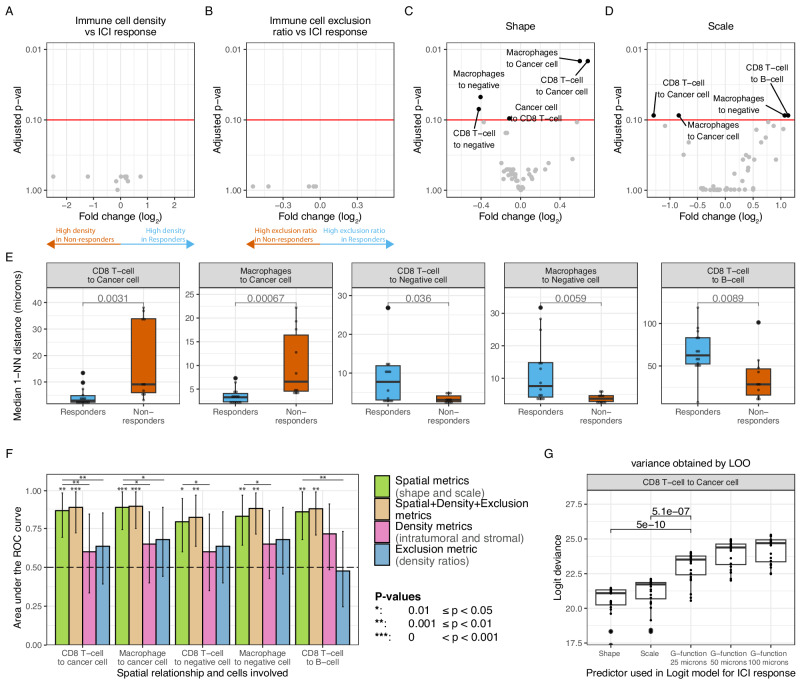
Association of spatial relationships with response to pre-operative ipilimumab+nivolumab in urothelial cancer. **A** Volcano plot showing the fold change on the intratumoral and stromal immune cell densities between response groups (x-axis) and statistical significance by t-test adjusted by multiple hypothesis testing (*y*-axis). **B** Volcano plot showing the fold change on the exclusion ratio (ratio between stromal and intratumoral immune cell density) between response groups (*x*-axis) and statistical significance by *t*-test adjusted by multiple hypothesis testing (*y*-axis). **C** Volcano plot showing the fold change on the shape parameter between response groups (x-axis) and statistical significance by *t* test adjusted by multiple hypothesis testing (*y*-axis). **D** Volcano plot showing the fold change on the scale parameter between response groups (*x*-axis) and statistical significance by *t*-test adjusted by multiple hypothesis testing (*y*-axis). **E** Median first nearest-neighbor distance distribution per response group as calculated by the associated shape and scale for the SRs that are significantly associated with response to ICI treatment (*n* = 14 independent responders and *n* = 10 independent non-responders) and statistical significance by a Mann*–*Whitney test. No adjustments were made for multiple comparisons. **F** ROC curve AUCs of the discriminative power of distinct TME parameters when predicting ICI response on *n* = 14 independent responders and *n* = 10 independent non-responders: all spatial parameters (green), all spatial parameters, density and exclusion ratio metrics (gold), all density metrics (pink), and exclusion ratio between stroma and tumor density (blue). Confidence intervals (error bar of each barplot) denote the 95% confidence interval as estimated by bootstrapping the 24 samples 500 times as implemented in pROC. The lines denote whether a statistical significance on the associated AUC was achieved. Significance symbols above bar plots denote whether the AUC is significantly different from AUC = 0.5 as assessed by a two-sided Mann–Whitney test. Significance symbols between bar plots denote whether the ROC-AUC-spatial (green) is significantly greater than the ROC-AUC-spatial-density-exclusion (gold) or ROC-AUC-density (pink) or ROC-AUC-exclusion (blue). Statistical significance between ROC curves was assessed by re-calculating AUCs by bootstrapping each ROC plot 500 times, and significance was assessed by a one-sided *t* test as implemented in pROC. Exact p-values are reported in the Source Data file. No adjustments were made to correct for multiple comparisons. **G** Logistic regression deviance of a univariate logistic regression model predicting ICI response (*n* = 14 independent responders and *n* = 10 independent non-responders) using as a predictor the shape, the scale, or the G-function evaluated at different thresholds (G-AUC-T). Variability on the AIC was evaluated by leave-one-out cross-validation on the 24 samples and significance was tested by Student’s *t* test. No adjustments were made for multiple comparisons. The box plots in each panel show the middle 50% of the data, with the box itself representing the median and the interquartile range (IQR) between the 25th and 75th percentiles. The whiskers extend from the box to the furthest data points within 1.5 times the IQR from the median. Unless otherwise stated, all statistical tests were two-sided. Significance symbols: *p < 0.05, **p < 0.01, ***p < 0.001. ICI: Immune checkpoint inhibitor; 1-NN: first nearest neighbor; ROC: Receiver operating characteristic; AUC: area under the curve; TME: tumor microenvironment; G-AUC-T: G-function evaluated at a threshold T; T: threshold; logit: logistic regression; LOO: leave-one-out cross validation. Source data are provided with this paper.

Multiplex immunofluorescence data are usually summarized as cell type fractions or immune cell density. We quantified the density of T cells, B cells, and macrophages in both the tumor and stromal compartments. However, we found no significant differences between response groups (Fig. 4A), indicating no differences between response groups in immune cell abundances in either the tumor and stromal compartments. In addition, immune cells spatially distribute following configurations of immune phenotypes^30^, being Excluded (high immune cell abundance in the stroma), Inflamed (high immune cell abundance in the tumor), and Desert (low immune cell abundance in the tumor and stroma). We quantified exclusion ratios (ratio between stromal and intratumoral immune cell density) and used them as a proxy of immune phenotypes for each immune cell. However, again we found no significant associations with treatment response (Fig. 4B), suggesting similar immune cell configurations in the response groups.

Motivated by the invariance between immune cell abundances and density ratios in the TME between response groups, we investigated whether spatial relationships derived from the TME were predictive of response to ICI combination treatment. We first investigated whether the SRs of all pairwise cell types, characterized by the Weibull parameters (shape and scale), were associated with clinical response (Fig. 1F). After correction for multiple hypothesis testing, we identified nine SRs that were associated with clinical response (FDR < 0.10) for the Weibull parameters shape and scale (Fig. 4C, D). The association between G-AUC-T and response using a rank-based statistic strongly depended on the selected value of the threshold, with the fold change decreasing with increasing values of the threshold (T) (Supplementary Fig. 6). When selecting a low threshold value (*T* = 25 μm), we found no significant associations between SRs quantified by G-AUC-25 and response (Supplementary Fig. 6A). Upon increasing the threshold value (*T* = 50 μm), we found three associations between SRs quantified by G-AUC-50 and response (Supplementary Fig. 6B), of which two were also identified using the Weibull parameters and one SR (FoxP3^+^ T cell to negative cell) was trending but not significant (FDR_scale_ = 0.21, FDR_shape_ = 0.11) using the Weibull parameters (Supplementary Figs. 6, 7).

To guide interpretation, we computed, for each SR significantly associated with clinical response to combination immunotherapy and each patient, the median 1-NN distances (Fig. 4E) and compared them using a rank-based statistic. Median distances exhibit a non-linear relationship with the shape and scale parameters (as indicated in Eq. 6) but provide enhanced clarity in the interpretation of our findings. In responding tumors, the distances from either CD8^+^ T cells or macrophages to the closest cancer cells were smaller than in non-responders (median 1-NN distance CD8^+^ T cell to cancer cell, responders = 4 ± 3μm, non-responders = 18 ± 15μm; Macrophage to cancer cell, responders = 4 ± 2μm, non-responders = 10.2 ± 7μm). Conversely, responding tumors had the largest 1-NN distances for the SR from CD8^+^ T cells or macrophages to the closest negative cell (median 1-NN distance CD8^+^ T cell to negative cell, responders = 9 ± 8μm, non-responders = 3 ± 1μm; Macrophage to negative cell, median 1-NN distance responders = 12 ± 10μm, non-responders = 4 ± 1μm). Despite the clear differences in the associated median 1-NN distances, the G-function approach did not identify the associations of the SRs with response at a low threshold (*T* = 25 μm, Supplementary Fig. 3A) nor the associations of the SRs involving CD8^+^ T cells and response at a higher threshold (*T* = 50 μm, Supplementary Fig. 3B, FDR = 0.14 and FDR = 0.20 for CD8^+^ T cell to cancer cell and CD8^+^ T cell to negative cell, respectively). Furthermore, non-responding tumors were characterized by small distances from CD8^+^ T cells to B cells (median 1-NN distance CD8^+^ T cell to B cell, responders = 66 ± 27 μm, non-responders = 36 ± 27 μm) when compared to responding tumors. We identified an association between the SR from cancer cells to CD8^+^ T cells and response with a small fold change for the shape parameter (|FC_shape_ | = 0.11, FDR_shape_ = 0.09), pointing to a difference in distribution that was not detected in terms of median 1-NN distances (Supplementary Figs. 7A-E, p = 0.98), suggesting that this may well be a false positive. Lastly, the single SR biomarker identified by the G-function approach at 50 μm but not at a 25 μm threshold (FoxP3 T cell to negative cell, FDR_G-AUC-25_ = 0.12, FDR_G-AUC-50_ = 0.04, Supplementary Fig. 6 and Supplementary Fig. 7J) that was not identified by the Weibull approach was trending (Supplementary Fig. 7I, FDR_shape_ = 0.11) and showed relative differences in the associated median 1-NN distances (Supplementary Fig. 7F).

In contrast to the SRs, the immune cell density and exclusion ratios were not associated with response. We confirmed, using simulated data, that density affects SRs between rare (e.g., immune to immune cells) but not between abundant and rare cell types (e.g., cancer to immune cells) (Supplementary Note 1). To further confirm independence between density and SR metrics in the predictive setting, we compared the predictive power for clinical response of the SR Weibull parameters (shape and scale) and their associated relevant density and exclusion metrics. The comparisons were made for each SR that was significantly associated with treatment response. For example, the SR from CD8^+^ T cells to cancer cells was associated with response (FDR_shape_= 0.01, FDR_scale_ = 0.09). We compared its predictive power with the CD8^+^ T cell density (intratumoral and stromal) and the exclusion ratio of CD8^+^ T cells. For this comparison, we employed a logistic regression model and the resulting area under the ROC curve (AUROC). The AUROCs for the five SR associations (depicted in Fig. 4E) and the associated density and exclusion metrics are shown in Fig. 4F. No density or exclusion metric reached significance as all 95% CI of the associated AUROCs included AUROC = 0.5. In contrast, all the SR Weibull parameters reached significance with AUROC values around 0.8 and 95% CI that do not include AUROC = 0.5 (Table 2, Supplementary Fig. 8), highlighting the superior predictive power of the SR metrics. Lastly, we tested whether adding density and exclusion ratio as covariates to the SR-based model improved the performance, but this was not the case (Fig. 4F, Table 2).

**Table 2:** Predictive power of logistic regression models as measured by the ROC-AUC for logistic regression models trained using only spatial metrics (shape, scale), density metrics (intratumoral and stromal density), exclusion ratio metrics (ratio between stromal and intratumoral density), and all metrics (all the metrics above)

Spatial relationship	Features included in model	AUC (95% CI)	P-value against Spatial ROC-AUC
CD8^+^ T cell to cancer cell	Spatial (shape and scale)	0.86 (0.70, 0.98)	–
CD8^+^ T cell to cancer cell	Density (Intratumoral CD8^+^ T cell density and Stromal CD8^+^ T cell density)	0.6 (0.34–0.85)	0.005
CD8^+^ T cell to cancer cell	Exclusion ratio (ratio between Stromal CD8^+^ T cell density and Intratumoral CD8^+^ T cell density)	0.64 (0.40−0.86)	0.02
CD8^+^ T cell to cancer cell	All TME features (spatial + density + exclusion ratio)	0.89 (0.74–0.99)	0.79
CD8^+^ T cell to negative cell	Spatial (shape and scale)	0.79 (0.59–0.95)	–
CD8^+^ T cell to negative cell	Density (Intratumoral CD8^+^ T cell density and Stromal CD8^+^ T cell density)	0.60 (0.34–0.84)	0.03
CD8^+^ T cell to negative cell	Exclusion ratio (ratio between Stromal CD8^+^ T cell density and Intratumoral CD8^+^ T cell density)	0.64 (0.39–0.86)	0.08
CD8^+^ T cell to negative cell	All TME features (spatial + density + exclusion ratio)	0.82 (0.63–0.96)	0.73
Macrophage to cancer cell	Spatial (shape and scale)	0.89 (0.73–0.99)	–
Macrophage to cancer cell	Density (Intratumoral Macrophage density and Stromal Macrophage density)	0.65 (0.41–0.86)	0.02
Macrophage to cancer cell	Exclusion ratio (ratio between Stromal Macrophage density and Intratumoral Macrophage density)	0.68 (0.46–0.88)	0.03
Macrophage to cancer cell	All TME features (spatial + density + exclusion ratio)	0.89 (0.74–0.99)	0.76
Macrophage to negative cell	Spatial (shape and scale)	0.83 (0.64–0.96)	–
Macrophage to negative cell	Density (Intratumoral Macrophage density and Stromal Macrophage density)	0.65 (0.41–0.86)	0.04
Macrophage to negative cell	Exclusion ratio (ratio between Stromal Macrophage density and Intratumoral Macrophage density)	0.68 (0.45–0.89)	0.08
Macrophage to negative cell	All TME features (spatial + density + exclusion ratio)	0.87 (0.71–0.99)	0.92
CD8^+^ T cell to B cell	Spatial (shape and scale)	0.86 (0.68–0.98)	–
CD8^+^ T cell to B cell	Density (Intratumoral B cell density and Stromal B cell density)^a^	0.72 (0.48–0.91)	0.16
CD8^+^ T cell to B cell	Exclusion ratio (ratio between Stromal B cell density and Intratumoral B cell density)	0.48 (0.23–0.72)	0.008
CD8^+^ T cell to B cell	All TME features (spatial + density + exclusion ratio)	0.88 (0.70–0.99)	0.58

*P*-value against spatial ROC-AUC denotes the difference between each model and the model trained spatial metrics from the associated SR by bootstrapping 500 times (testing whether the AUC is greater). Confidence intervals of ROCs were estimated by bootstrapping samples 500 times. Error bars denote the 95% confidence interval as estimated by bootstrapping the 24 samples 500 times as implemented in pROC. Significance was estimated by evaluating whether the ROC-AUC is significantly greater than the ROC-AUC-spatial (ROC plot built with spatial parameters) by re-calculating AUCs by bootstrapping each ROC plot 500 times, and significance was assessed by a one-sided t-test as implemented in pROC. No adjustments were made to correct for multiple hypothesis testing.

*ROC* receiver operating characteristic, *AUC* area under the curve, *SR* spatial relationship, *CI* confidence interval.

^a^CD8+ T cell density was not included in the model as it was already included in the density models of the SRs CD8+ T cell to Cancer cell and CD8+ T cell to negative cell.

Next, we compared different SR metrics derived from the 1-NN distance distribution (shape, scale, G-AUC-T at different T) for their ability to describe clinical response. To do so, we compared the Weibull parameters to the G-AUC-T metrics in the predictive setting. Specifically, we compared the logistic regression deviance, in which lower values indicate better model fits. We did so for the six SRs that were found to be significantly associated with response. The SR from CD8^+^ T cells to cancer cells model showed that the shape or the scale parameters scored significantly better than the G-AUC-T trained models for *T* = 25, 50, and 100 (Fig. 4G). For the remaining SRs significantly associated with clinical response (Fig. 4C, D), we observed that the models trained using Weibull parameters (shape and scale) outperformed the models trained using G-function parameters, except for the SR from macrophages to negative cells and from CD8 T cells to B cells where G-AUC-25 performed similarly as in the Weibull approach (Supplementary Fig. 9).

In summary, we observed that mIF-derived spatial relationships in the TME hold superior predictive power for clinical response compared to immune cell density or immune phenotypes. Our results convincingly demonstrate that the Weibull parameters (shape and scale) are superior to the G-function metrics (G-AUC-T) in predicting clinical response to combination checkpoint therapy.

### Validation of spatial relationships biomarkers of ICI response in a cohort of head and neck cancer

We tested whether our spatial biomarkers also predicted response in other cancer types. We used a cohort of head and neck squamous cell carcinoma (HNSCC) patients from the IMCISION trial^28^ to validate our findings. A subset of 25 IMCISION patients was treated with pre-operative ipilimumab+nivolumab (similar to NABUCCO), and successfully provided tumor sample profiling with the same mIF antibody panel as the UC cohort (Fig. 1G, Table 1).

**Fig. 5:**
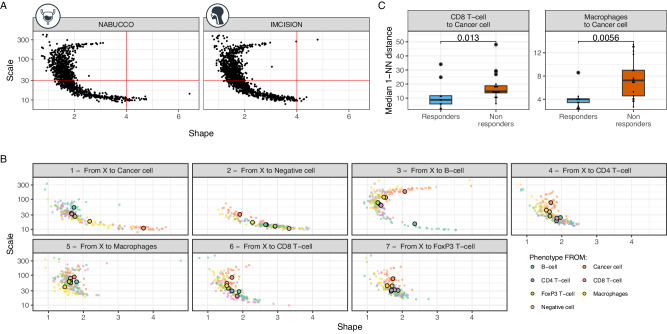
Validation of spatial relationship biomarkers of ICI response in an independent cohort of pre-operative ipilimumab+nivolumab in head and neck cancer. **A** Scale vs. shape SR parameters fitted on the 1-NN distribution for the 25 samples and the 7 × 7 cell type combinations obtained in the head and neck cancer data (right, IMCISION) and UC data (left, NABUCCO). **B** Scatter plot of the scale-shape parameter space by neighbor cell type (cell to) obtained in the IMCISION trial data. Cohort averages for their associated spatial parameters are highlighted as big dots for each cell type-cell type combination. **C** Median first nearest-neighbor distance distribution in head and neck cancer samples per response group (*n* = 9 independent responders and *n* = 16 independent non-responders) as calculated by the associated shape and scale for the SRs that significantly (FDR < 0.04) associated with response in UC and statistical significance by a Mann–Whitney test. No adjustments were made for multiple comparisons (adjustments for multiple hypothesis testing were done on the shape and scale parameters space in Supplementary Fig. 11). The box plots in each panel show the middle 50% of the data, with the box itself representing the median and the interquartile range (IQR) between the 25th and 75th percentiles. The whiskers extend from the box to the furthest data points within 1.5 times the IQR from the median. Unless otherwise stated, all statistical tests were two-sided. Icons from (A, B, F, G) were adapted from bioIcons (cancerous-cell-1, lymphocytes-4, macrophage, t-lymphocyte, b-lymphocyte, fibroblast-1 licensed under CC-BY 3.0 Unported by Servier), flaticon.com (bladder icon, https://www.flaticon.com/free-icon/bladder_1453578; head neck icon, https://www.flaticon.com/free-icon/injection_4418017). SR: spatial relationship; ICI: immune checkpoint inhibitors; 1-NN: first nearest neighbor; UC: urothelial cancer. Source data are provided with this paper.

We first compared the SR parameter space in HNSCC (Supplementary Data 3) with that of the UC cohort. We observe the same C-shape distribution in the shape-scale space as we observed in UC (Fig. 5A). Second, we found a high concordance between NABUCCO and IMCISION for the shape and scale population averages across pairwise SRs between all cell types (Fig. 5B, Supplementary Figs. 10A, D). These results suggest that the distance between cell types follows a characteristic pattern preserved across these two cancer types. For instance, similar behavior of B cell to B cell 1-NN distances compatible with the “Low scale, High shape” behavior was observed in HNSCC.

Next, we evaluated whether the SR biomarkers of ICI response identified in UC were also predictive of reaching a major pathological response upon combination ICI in the HNSCC cohort. The validation was assessed for the strongest biomarkers (FDR < 0.04) to maximize the likelihood of validation, which involved the SRs *CD8*^+^ *T cells to cancer cells* and *Macrophages to cancer cells* from Fig. 4C-D. Both SRs showed the same direction of association with response in HNSCC (Fig. 5B) and, importantly, showed a statistically significant association with response after multiple testing correction: CD8^+^ T cells to cancer cells (FDR_shape_=0.045) and macrophages to cancer cells (FDR_shape_=0.0076, FDR_scale_ = 0.00094) (Supplementary Fig. 11A, B), which matches with the spatial proximity behavior (lower 1-NN distances) identified in the responding UC tumors (Fig. 4E). We confirmed the earlier established superiority of the SR metrics over density metrics by showing that, in the HNSCC cohort, immune cell density was not associated with response (Supplementary Fig. 11C), except for stromal macrophage and CD8^+^ T cell densities.

In conclusion, the TME spatial biomarkers for pathological response to ICI combination treatment in UC validated in an HNSCC cohort, suggesting that the SRs between CD8^+^ T cells and macrophages to cancer cells could be an important context-independent biomarker for clinical response to ipilimumab+nivolumab.

## Discussion

Advances in ICI have resulted in pembrolizumab (anti-PD1) becoming the second-line standard of care for advanced UC^3^, and avelumab (anti-PD-L1) as the standard of care for maintenance after chemotherapy treatment^31^. Results from pre-operative clinical trials show that patients can have a pathological complete response to only two or three cycles of immunotherapeutic treatment^9,10,32,33^. These promising clinical results need biomarkers that stratify individual patients and improve our understanding of the immunological background of (non-)response. In this study, we provided a comprehensive quantitative exploration of the, thus far, poorly characterized SRs in the UC TME. We show the potential for clinical utility in predicting the response to pre-operative combination ICIs and provide a quantitative basis for follow-up research.

We found an association between the proximity of the SR from CD8^+^ T cells to cancer cells and response in UC and confirmed that this association also holds in HNSCC. In contrast, no differences between response groups in CD8^+^ T cell density were found, revealing that abundance alone is likely insufficient to explain treatment response. Tumors with an immune excluded phenotype exhibit an enrichment of CD8^+^ T cells at the stroma due to mechanisms preventing T cells from reaching the tumor. Quantifying immune phenotypes is not trivial as distinct patterns of exclusion and topography exist^17,34^. In our study, we used exclusion ratios and densities as a proxy to estimate immune phenotypes, but we found no difference between response groups. In contrast, our SR parameters served as a distance metric that objectively quantifies proximity differences between CD8^+^ T cells and cancer cells between response groups. Therefore, while abundances or ratios between abundances of CD8^+^ T cells were insufficient to explain response to combination ICIs, a more complex quantification of their relative spatial distribution in relation to cancer cells was a more informative way to describe their behavior within a tumor. Our observations suggest that therapeutic strategies that enhance CD8^+^ T cell migration closer to cancer cells may overcome resistance to ICI. These results align with the observation that immunosuppressive mechanisms, such as TGF-beta signaling, are associated in UC^10,12^ with a CD8^+^ T cell excluded phenotype and resistance to ICI^35^. Similar results have been reported in the ICI context for melanoma, in which responding tumors to different ICI treatments were characterized using a 1-NN statistic by proximity between proliferating antigen-experienced CD8^+^ T cells (CD45RO^+^Ki67^+^) and their closest cancer cell^23^. Moreover, in gynecological and non-small cell lung cancer, the SR between tumor-infiltrating lymphocytes (TIL) and non-TILs (e.g., cancer cells) demonstrated its utility for clinical outcome prediction in an ICI cohort^24^, which is compatible with our observations in UC and HNSCC.

We found that the proximity of macrophages to cancer cells was positively associated with response in the UC and HNSCC cohorts. Interpretation of this candidate biomarker warrants further investigation due to the plasticity and potential pro- or anti-tumorigenic behavior of macrophages^36^, which results in macrophage subtype heterogeneity not covered by our mIF antibody panel. Literature in the ICI context suggests that macrophages can express PD-L1 and PD-1^37^ but can also prevent T cells from reaching cancer cells^38^. Data from pancreatic cancer suggest that anti-tumorigenic macrophages (M1-macrophages) are closer to cancer cells than pro-tumorigenic macrophages (M2 macrophages)^39^, which indicates that our proximity signal between macrophages and cancer cells in responding tumors may originate from an M1-type macrophage lineage. In locally advanced esophageal squamous cell carcinoma treated with chemoradiotherapy and SHR-1210 (anti-PD-1 ICI), a prognostic signal using the 1-NN statistic median reported PD-L1^+^ tumor cells closer to PD-L1^-^ macrophages associated with a better OS after treatment^25^. Lastly, non-responding tumors were associated with close proximity between B cells surrounding CD8^+^ T cells, which is in line with the high baseline expression of genes involved in B cell signaling we found in non-responding UC tumors in NABUCCO^10^.

We compared the spatial and density metrics’ predictive power to corroborate the SR metrics’ importance. Our results show a superior predictive power for SR metrics and enhance the limited view that count-derived data, such as density or exclusion ratios, provide of the TME. Furthermore, we compared the SR quantifications on our Weibull parameters with the conventional G-function. While both are based on the 1-NN distance statistic, we showed that the G-function dependence on a distance threshold (T) reduced its utility for group comparisons because the associated G-function’s range of values, variance, and predictive power was threshold dependent. Besides, due to the heterogeneity in the G-function evaluations across cell-cell pairwise relationships, there is no unique optimal threshold that maximizes differences between clinical groups of interest for all SRs. Therefore, optimization methodologies for the threshold of choice depend on the SR and cohort, potentially leading to under- or over-fitting and generalization issues. Earlier work on the G-function metric usage for pancreatic cancer grade prediction reported that a single threshold evaluation cannot model all the inherent signals from the data^26^. A higher predictive power could only be achieved by discretizing the G-function at multiple thresholds, which limits its interpretability and utility because of an increased number of summary parameters^26^. On the other hand, our Weibull parameters (shape and scale) allowed for an invariant summary of the SRs without any threshold, which was achieved by, instead of having an empirical summary or discretizing it, modeling all its inherent structure using a curve-fitting approach. Furthermore, the mixed model methodology allowed us to smooth the data and model the parameter variance at a cohort level, making it more suitable for group comparisons when correlating them with clinical phenotypes of interest because of the reduced leverage of outlier samples^40^.

The validation of our TME candidate biomarkers for pre-operative combination ICIs response identified in UC indicates biologically relevant SR differences consistent across cancer types. Crucially, despite HNSCC being a different organ and morphologically distinct tumor type, showing variability within their biopsy locations (oral cavity, oropharynx), using our proposed spatial approach, we observed similar average SR distances in both tumor types. A combination of pathological complete response and near-complete response defined response for exploratory analyses in IMCISION (HNSCC). However, in NABUCCO (UC), treatment response was defined as a pathological complete downstaging at the time of surgery. The response rate in IMCISION was lower than in NABUCCO (36% vs. 58%, p = 0.04), thus decreasing the statistical power to quantify differences between response groups. Despite these differences and the relatively small sample sizes (24 and 25 for NABUCCO and IMCISION, respectively), the translatability of our findings on the associations between the SR parameters identified in our UC cohort and treatment response in the HNSCC cohort is promising.

Limitations of our study include the number of antibody markers profiled in mIF data, which restricted the types of cells we could detect. Transurethral resections provide a superficial spatial sampling of the whole TME architecture, therefore allowing for limited profiling of the tumor margin, which is known to contain a higher abundance of immune cells in UC^34^ compared to intratumoral tissue. Nevertheless, the literature suggests that transurethral resection (TUR) material in UC is representative of the whole UC tumor spatial heterogeneity in ~58-73% of cases at an immune cell density level^41^, but their associated SRs remain yet unexplored. Limitations to our methodological framework include quantifying SRs by studying only the first nearest neighbor and not beyond. While considering higher-order neighbors could facilitate exploring more distant spatial patterns, the trade-off involves a compromise in the interpretability of the data. Network or graph-based approaches would allow for a broader spatial representation of the TME. However, these topology-based methods usually ignore distances and require more complex SR representations. Furthermore, combinations of samples and pairwise cell type SRs involving noisy distance distributions, such as SRs derived from lowly-populated cells (e.g., FoxP3^+^ T cells in a subset of UC samples), are excluded from the analysis only when convergence is not reached in the mixed model fitting. However, only 2% of our SRs (24 out of 1176 SRs) were rejected for this reason. While this might have consequences in associations with clinical outcomes of interest (e.g., clinical response), such rare cell types SRs lack robustness. Lastly, our sample sizes are relatively small, and our results warrant further validation in independent and larger cohorts.

In short, our study provides a systematic framework to quantify SRs. It demonstrates that SRs provide a complementary summary of the TME outperforming count-derived metrics, such as density, for identifying biomarkers with a clinical utility. Our results reveal proximity between CD8^+^ T cells to cancer cells and macrophages to cancer cells as candidate biomarkers for response to pre-operative combination ICIs, which have been thus far unexplored and provide a complementary view of the TME that warrants further investigation.

## Methods

### Study oversight

The studies from this manuscript received approval from the institutional review board of the Netherlands Cancer Institute - Antoni van Leeuwenhoek Hospital. The execution of these studies strictly adhered to the protocols and Good Clinical Practice Guidelines outlined by the International Conference on Harmonization, along with the principles established in the 1964 Declaration of Helsinki. Approval for the trial protocols and any subsequent amendments was obtained from the Medical Research Ethics Committee of the Netherlands Cancer Institute—Antoni van Leeuwenhoek Hospital (MREC AVL, https://english.ccmo.nl/mrecs/accredited-mrecs/mrec-netherlands-cancer-institute-the-antoni-van-leeuwenhoek-hospital). Before enrolling in the clinical trials, all participating patients provided written informed consent to partake in the studies.

### Urothelial cancer study population and treatment (NABUCCO trial)

Twenty-four pre-treatment Urothelial Cancer (UC) samples from the NABUCCO trial (NCT03387761, Cohort 1) were used for analyses. In the trial patients underwent a combination treatment (2 or 3 cycles) of ipilimumab (anti-CTLA-4) and nivolumab (anti-PD-1) prior to surgical resection. The trial cohort consisted of high-grade stage III muscle-invasive urothelial cancer (cT3-4aN0M0 or cT1-4aN1-3M0). Details of the trial are reported^10^.

Response to treatment was evaluated by pathological response assessment on radical surgery. Tumors with a complete pathological response (ypT0N0) or residual disease (<=ypT1N0) were classified as responders (*n* = 14), and tumors with a >=ypT2N0 were classified as non-responders (*n* = 10).

### Head and Neck squamous cell carcinoma population and treatment (IMCISION trial)

Thirty-one head and neck squamous cell carcinoma (HNSCC) tumor samples of multiple subsites (oral cavity *n* = 27, oropharynx, *n* = 4) were obtained from the IMCISION trial (NCT03003637). Patients underwent either two cycles of nivolumab (Arm A, *n* = 6) or a combination treatment of 2 cycles of ipilimumab (anti-CTLA-4) and nivolumab (anti-PD-1) (Arm B, *n* = 25) prior to surgical resection. The trial cohort consisted of HNSCC tumors with a histological grade T2‒T4N0‒N3b and metastatic grade M0 primary or recurrent of mostly HPV-negative head and neck squamous cell carcinoma (HPV negative, *n* = 23; HPV positive, *n* = 2). Details of the trial can be found elsewhere^28^. Only samples from Arm B (*n* = 25) were analyzed in this manuscript.

Response to treatment was evaluated by pathological response assessment on surgery and by comparison of tumor cells decrease from baseline to on-treatment samples^28^. Tumors with <=10% tumor cell percentage (TCP) at surgery and a decrease of 90-100% in tumor cells from baseline to on-treatment were classified as major pathological responders (MPR, *n* = 9); tumors with <=50% TCP at surgery and a decrease of 50-89% in tumor cells from baseline to on-treatment were classified as partial pathological responders (PPR, *n* = 1); else tumors were classified as no pathological responders (NPR, *n* = 15). Patients with an MPR were classified as Responders, and patients with a PPR or NPR were classified as Non-Responders.

### Multiplex immunofluorescence

Multiplex immunofluorescence (mIF) was performed on pre-operative baseline formalin-fixed paraffin-embedded (FFPE) tumor resections and assessed on an immune panel (DAPI, PanCK, CD8, CD3, FoxP3, CD20, CD68) as previously described for UC^10^ (NABUCCO) and HNSCC^28^ (IMCISION). The experimental protocol and data processing is reported elsewhere^10,28^.

Antibodies used for the NABUCCO trial dataset were CD3 (1/400 dilution, Clone P7, Cat RM-9107-S, ThermoScientific), CD8 (1/100 dilution, Clone C8/144B, Cat M7103, DAKO), CD68 (1/500 dilution, Clone KP1, M0814, Dako), FoxP3 (1/50 dilution, Clone 236 A/47, Cat ab20034, Abcam), CD20 (1/500 dilution, Clone L26, cat M0755, Dako), PanCK (1/100 dilution, Clone AE1AE3, Cat MS-343P, Thermo Scientific). Antibodies used for the IMCISION trial dataset were CD3 (clone SP7, ThermoScientific, CatalogNo: RM-9107-S, LotNo: 9107S1805A), CD8 (clone C8/144B, DAKO/Agilent, CatalogNo: M7103, LotNo: 20048132), CD68 (clone KP1, DAKO / Agilent, CatalogNo: M0814, LotNo: 20040389), FoxP3 (clone 236 A/47, DAKO/Agilent, CatalogNo: ab20034, LotNo: GR3220121-1), CD20 (clone L26, DAKO / Agilent, CatalogNo: M0755, LotNo: 20038880), PanCK (clone AE1AE3, Thermoscientific, CatalogNo: MS-343P, LotNo: 343P1205H).

Upon mIF profiling, cells were segmented by marker positivity, and classified as Cancer cells (PanCK^+^), CD8 T cells (CD3^+^CD8^+^), FoxP3 T cells (CD3^+^FoxP3^+^), CD4 T cells (CD3^+^CD8^-^FoxP3^-^), Macrophages (CD68^+^) and B cells (CD20^+^).

Multiplex immunofluorescence in IMCISION was assessed as in NABUCCO and the experimental protocol is published in the original manuscript^28^. To ensure consistency in the mIF spatial data between the HNSCC and UC cohorts, including similar tumor purity, for each sample from IMCISION we aligned analysis methods by discarding stromal tissue residing beyond 150 μm distance from the tumor tissue by filtering out all cells classified as belonging to the ‘Stroma compartment’ (by segmentation) if their closest cancer cell lay beyond 150 μm by using the nncross method from *spatstat*.

The data subjected to downstream analysis represented the position of each cell in the tissue (x- and y-coordinates of the nuclei) and its corresponding cell type.

### Segmentation of tumor and stroma compartments

To segment the tumor and stroma regions from each tissue, we first split each individual tissue island from each sample biopsy using dbscan v1.1-6 (density-based spatial clustering of applications with noise) by setting the size of the epsilon neighborhood to 300 and the minimum number of points in the epsilon neighborhood to 50 (Supplementary Fig. 1A). Each tissue island was named foci.

To segment the tumor and stroma compartments for each focus, we first computed the kernel density estimation (KDE) of the point pattern defined by cancer cells (KDE_tumor_), and by negative cells (KDE_negative_). The KDE was estimated as implemented by density in the stats v3.6.3 package. The smoothing bandwidth for the KDE was optimized using likelihood cross-validation as implemented in *bw.ppl* in spatstat v1.64-1. Then, the KDEs were normalized to their maximum value (KDE_tumor =_ KDE_tumor_ / max(KDE_tumor_)) to allow comparison between the KDE of the tumor and negative cells. To segment the tissue, for each position populated by a cell, we compared both KDEs, and classified them as “Tumor” when KDE_tumor_ > KDE_negative_ and as “Stroma” otherwise (Supplementary Fig. 1B-C).

### Calculation of tumor and stroma compartment areas

To compute the covered area by each segmented tissue compartment (“tumor” or “stroma”), we first computed the kernel density estimation (KDE) of the point pattern defined by the cancer cells (KDE_tumor_) and normalized by maximum KDE intensity. Then, to compute the area of the tumor compartment, we filtered out all the KDE pixels with a normalized intensity <0.1. We selected this threshold based on visual exploration for all cells. We then estimated the tumor compartment area as the aggregated area of all non-filtered pixels from KDE_tumor_ (thus with intensity >= 0.1).

To compute the total tissue area, we also computed the KDE_negative_ and normalized it by the maximum value. We then summed the KDE_tumor_ and KDE_negative_, and filtered out pixels with a normalized intensity <0.1. We then estimated the Total tissue area as the aggregated area of non-filtered pixels from (KDE_tumor_ + KDE_negative_) (intensity >= 0.1).

To estimate the area of the “stroma” compartment, we subtracted the “tumor area” from the “total tissue” area (Supplementary Fig. 1D). This process was performed for each foci.

### Spatial analysis: quantification of the first nearest neighbor (1-NN) distance distribution

The spatial relationships between all cells within the tumor microenvironment were studied using the first-nearest neighbor (1-NN) statistic as implemented in *spatstats*. In brief, the approach is studied from a reference cell type to a neighbor cell type. For each reference cell type (“cell type from*”*), the distance to the closest neighbor cell type (“cell type to*”*) was measured using *nndist* (Fig. 1E). Then, we constructed a histogram from the vector of 1-NN distances. We smoothed the distribution by sliding a 5-μm window across the 1-NN histogram and iteratively counting the frequency of the 1-NN distances for each micrometer. We normalized the distribution to achieve a unit area under the curve (AUC) by dividing for the numerical AUC. SRs were quantified using the data from the whole tissue slide (i.e., not making a distinction beween tumor and stroma compartments).

### Spatial analysis: fitting of Weibull distribution to the 1-NN distances vector

To summarize the 1-NN distance distribution, we fitted a Weibull distribution to the empirical probability density function (PDF), which is a 2-parameter distribution based on (the positive parameters) shape and scale, defined as:

1 $$f(x,\,\,b,\,a)=\frac{a}{b}{\left(\frac{x}{b}\right)}^{a-1}{e}^{-{(x/b)}^{a}}$$

Here, *b* denotes the scale, and *a* denotes the shape.

We implemented a methodology based on a functional data analysis approach to fit the Weibull distribution. First, to have an initial estimate of the distribution parameters for each patient (*n* = 24) and cell type-cell type combination (n = 49), we employed maximum likelihood estimation (MLE) using fitdist as implemented on fitdistrplus v1.1.3 package to have an initial estimate of the scale and shape parameters. Then, for each pairwise cell type relationship (cell type from vs. cell to), we implemented a non-linear mixed effect model (nlme v3.1-144) to fit a Weibull distribution on all patient samples, having the shape / scale intercept as fixed effects (fixed = *a* + *b* ~ 1) and allowing a random effect for the scale/shape on each sample (random = list{sample=pdDiag(*a* + *b* ~ 1)}) by modeling the correlation structure of the random effects a with diagonal positive-definite matrix. The nlme model was implemented by maximizing the restricted log-likelihood (method = ‘REML’), with the parameters set to their default values and the control values set as:

- 1000 maximum iterations for the optimization algorithm (maxIter = 1000).
- 200 maximum iterations for the optimization step which is inside the nlme optimization process (msMaxIter = 200).
- 1e−1 tolerance for the PNLS step convergence criterion (pnlsTol=1e−1).
- 1e−6 tolerance for the convergence criterion in nlme algorithm (tolerance = 1e−6).
- Nonlinear minimization optimizer (opt = “nlm”).

To filter out low-quality distributions that lead to non-convergence of the models, we sequentially filtered out samples based on the number of cells (*n*) from the reference cell type (nFROM) or neighbor cell type (nTO). First, we fitted a model with the data for the 24 samples. If convergence was not achieved, we filtered out all samples with fewer than 20 cells (nFROM < 20 or nTO < 20). If the model still did not converge, we repeated filtering samples with fewer than 50, 70, or 100 cells. The approach allowed us to model as much data as possible unless the goodness of fit was compromised. For 24 spatial relationships for which a data fit could not be carried out (2% of the total combinations of data points from 24 samples and 7 × 7 cell type pairwise relationships) in our cohort.

Because the positivity constraint for the Weibull distribution parameters (*b* > 0, *a* > 0) could not be optimally implemented using a constrained non-linear mixed effect model, we re-parameterized the shape and scale parameters to force the values to be positive. In brief, we re-parameterized the scale and shape employing new parameters *A* and *B*, which were unconstrained:

2 $$a=\frac{10}{1+{e}^{-A}}\,{{{{{\rm{with\; A}}}}}}:\,{{{{{\rm{unconstrained\; and}}}}}}\,{{{{{\rm{a}}}}}} \, \epsilon \, ({{{{\mathrm{0,10}}}}})$$

3 $$b=\frac{500}{1+{e}^{-B}}{{{{{\rm{with}}}}}}\,{{{{{\rm{B}}}}}}:{{{{{\rm{unconstrained}}}}}}\,{{{{{\rm{and}}}}}}\,{{{{{\rm{b}}}}}} \, \epsilon \, ({{{{\mathrm{0,500}}}}})$$

Here, *a* is the shape and *b* is the scale.

Unless otherwise stated, the SR parameters reported in the manuscript correspond to the ones calculated on the first-nearest neighbor distributions using the spatial distribution of the whole tissue slide (thus, not using data stratified by tumor or stroma).

Sources of the variability of the SR parameters were also quantified, as reported in Extended Methods.

### Spatial analysis: computation of the G-function

Alternatively to fitting a distribution to the 1-NN distance distribution, we computed the G-function, which is defined as the cumulative distribution function (CDF) of the first-nearest neighbor distance distribution:

4 $$G-{function} \, (r)={probability}(1-{NN}\,{distance} \, \le \, r)$$

The G-function was computed as implemented by *gest* in the spatstat package. Because our SRs were studied between different cell types, we used the multitype nearest-neighbor function G_ij_(r) as implemented in Gcross, which was calculated from the first nearest neighbor distances from a cell of type i to the nearest point of type j. Then, to summarize the G-function, we computed the Area Under the Curve (AUC) of the G-function, named G-AUC-T, at different thresholds T’s, which included 25, 50, and 100 μm (i.e., G-AUC-25, G-AUC-50, and G-AUC-100, respectively).

### Spatial analysis: Weibull-derived G-function

Using the properties of the Weibull distribution, we analytically constructed the Cumulative Distribution Function (CDF) using the shape and the scale parameters:

5 $${Analytical\; CDF} \, (r)=1-\exp (-{(r/b)}^{a})$$

Here, *b* and *a* denote the scale and shape, respectively, and *r* denotes the first nearest neighbor distance.

The Analytical CDF is analogous to the Analytical G-function. The AUC of the Analytical CDF was evaluated at different distance thresholds T’s, and referred to in the manuscript as Weibull-G-AUC-T.

### Comparison of discriminative power between spatial and density-related parameters

We compared the predictive power between ICI response groups of the spatial-related parameters (shape, scale), density-based parameters (intratumoral and stromal immune cell density) and exclusion-based parameters (ratio between stromal and intratumoral immune cell densities). First, for each set of parameters (e.g., spatial-related parameters), we trained a logistic regression model using glm to predict response. Then, a ROC curve was built using the logistic regression’s fitted values (probabilities) using pROC v1.17.0.1. For each ROC-AUC, we tested whether the AUC was significantly different from AUC = 0.5 using a two-sided Wilcoxon signed-rank test between cases and controls. We used a bootstrapped approach (*n* = 500) to estimate the confidence intervals of the ROC-AUCs and to test whether the ROC-AUCs from the spatial parameters were significantly greater than the ROC-AUCs from either the density or the exclusion ratio parameters.

### Comparison of discriminative power between Weibull-derived or G-function-derived parameters

Logistic regression deviance of Weibull parameters (shape, scale) and G-function parameters (G-AUC-T evaluated at different Ts) was evaluated by training univariate logistic regression (LR) models using data from each feature as a predictor, and clinical response labels as the dependent variable. Logistic regression deviance (logit deviance) was assessed, in which lower values denote better model fits. The uncertainty of the AIC was estimated by performing a leave-one-out (LOO) variant of the analysis. A two-sided student’s t-test tested statistical significance.

### Spatial biomarker validation in HNSCC

We validated our top SR biomarkers associated with clinical response in our UC cohort (NABUCCO). First, we selected the top two biomarkers identified using our pipeline in UC (FDR < 0.04 in either the shape or scale parameter, which yielded the SRs *CD8*^*+*^ *T cells to cancer cells* and Macrophages to cancer cells*)*. Second, we evaluated the shape and scale parameters for the biomarkers mentioned above between clinical response groups in the external HNSCC (IMCISION) cohort using a two-sided t-test and adjusted for multiple hypothesis testing using the Benjamini-Hochberg method. Then, we evaluated the median 1-NN distances using the analytical derivation from the shape and scale parameters:

6 $${Median} \, 1-{NN}\,{distance} \, ({shape},\, {scale})={scale} \,*\, {({{{{\mathrm{ln}}}}}2)}^{1/{shape}}$$

### Statistical analysis

Unless otherwise stated, a two-sided student’s t-test was used for group comparisons. We modeled density and count data in a logarithmic space. For spatial data, the shape of the Weibull parameters (*a*) was modeled on a non-logarithmic scale, and the scale (*b*) was studied on a logarithmic scale. Multiple hypothesis testing corrections were done using the Benjamini–Hochberg method. Unless otherwise noted, statistical significance was defined as *p* < 0.05 and False Discovery Rate (FDR) < 0.10 (10%), and all statistical tests were two-sided. All statistical analyses were performed in R 3.6.3. The following packages were used in this study:

- Spatstat 1.64^29^
- Dplyr 1.0.4
- Fitdistrplus 1.1.3
- Patchwork 1.1.1
- Survival 1.3.24
- ComplexHeatmap 2.2
- Circlize 0.4.12
- Glmnet 4.1.1
- RColorBrewer 1.1.2
- Nlme 3.1.144
- Spastat 1.7.0
- Ggpubr 0.4.0
- Ggrepel 0.9.1
- Plyr 1.8.6
- Tidyverse 1.3.0
- Ggplot 2.3.3.3
- Tibble 3.0.6
- ggrastr version 1.0.1
- pROC 1.17.0.1

### Reporting summary

Further information on research design is available in the Nature Portfolio Reporting Summary linked to this article.

### Supplementary information

Supplementary Information

Peer Review File

Description of Additional Supplementary Files

Supplementary Data 1

Reporting Summary

### Source data

Source data

## Data Availability

The multiplex immunofluorescence data consisting of the spatial coordinates and immune cell types linked with the clinical data used for this manuscript are available from the authors upon request within the restrictions of the informed consent. The institutional review board of the Netherlands Cancer Institute will review every request. After approval, the researcher will need to sign the Netherlands Cancer Institute data access agreement. Multiplex immunofluorescence derived data (spatial parameters and densities) are made available as Supplementary Data 1 and Source Data files to reproduce the findings from the manuscript. All other data are available in the article and its Supplementary files or from the corresponding author upon request. Source data are available with this paper. Source data are provided with this paper.

## Code availability

Code to reproduce the main findings are available in a Github repository: https://github.com/tropicalberto/nabucco_spatial_manuscript.
