# Supplementary material for: Spatial relationships in the urothelial and head and neck tumor microenvironment predict response to combination immune checkpoint inhibitors
Source: Nat Commun. 2024 Mar 21;15:2538. doi: 10.1038/s41467-024-46450-1 (PMC10957922; doi:10.1038/s41467-024-46450-1)
Supplement: Supplementary file 1 — Supplementary Information [file 41467_2024_46450_MOESM1_ESM.pdf]

Title

Spatial relationships in the urothelial and head and neck tumor microenvironment predict response to combination immune checkpoint inhibitors

Supplementary Information

Supplementary Figures

Supplementary Figure 1

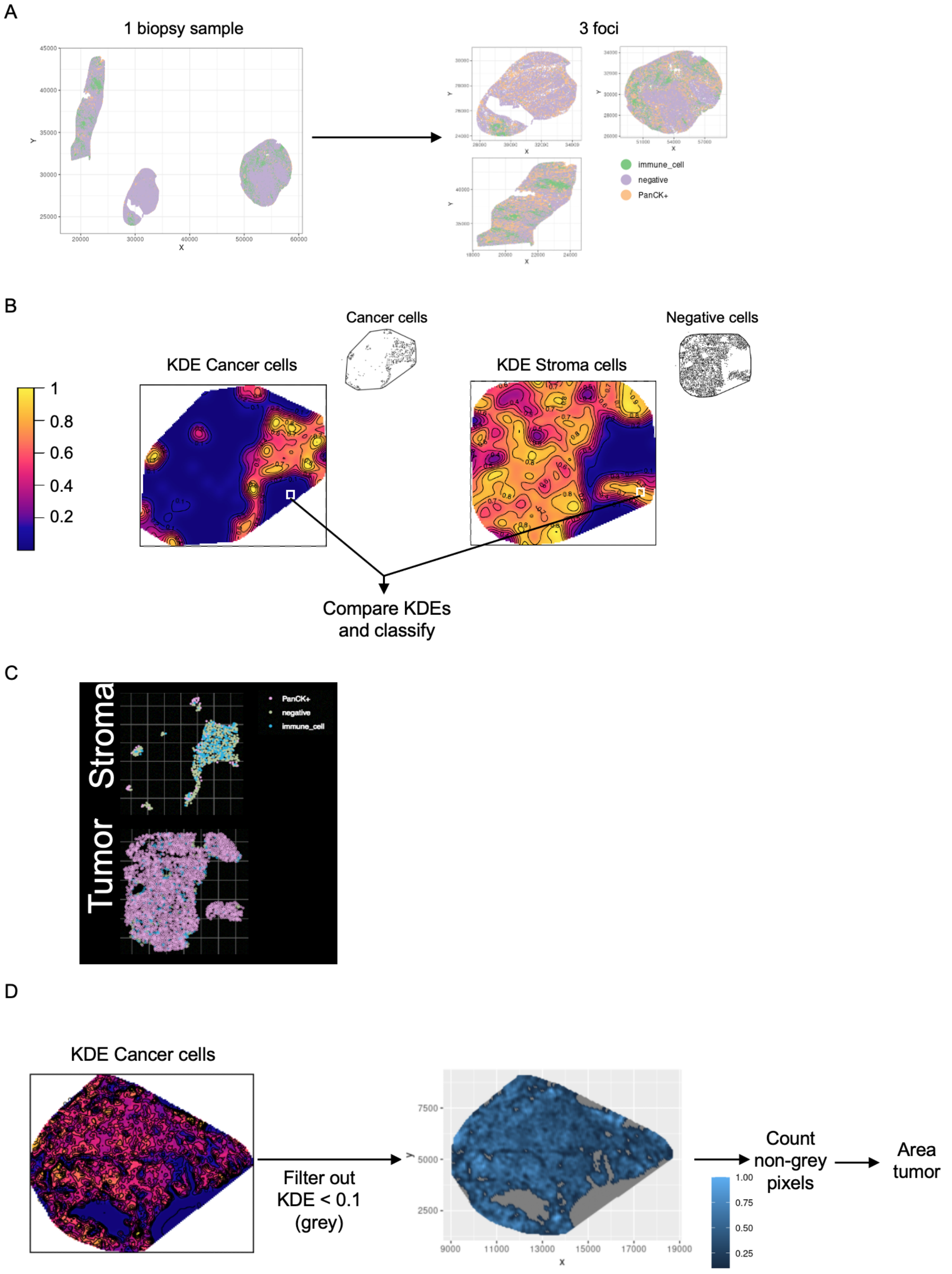
A
1 biopsy sample
3 foci
immune_cell
negative
PanCK+
B
Cancer cells
Negative cells
KDE Cancer cells
KDE Stroma cells
1
0.8
0.6
0.4
0.2
Compare KDEs
and classify
C
Stroma
Tumor
PanCK+
negative
immune_cell
D
KDE Cancer cells
Filter out
KDE < 0.1
(grey)
Count
non-grey
pixels
Area
tumor
1.00
0.75
0.50
0.25

Supplementary Figure 1. **Segmentation of tumor and stroma compartments and tissue area assessment.**
(A) Visual representation of a biopsy tissue slide (left) that contains multiple tissue islands. Each tissue island was identified using the dbscan algorithm and named foci (right).
(B) Tumor and stroma regions segmentation by comparison of the Kernel density estimation (KDE) computed using only cancer cells (left) or stroma cells (right). Prior to segmentation, KDEs were normalized by the maximum value.
(C) Example of tumor and stroma classifications. Cells in the top and bottom panels are classified as being in the stroma or the tumor compartment, respectively. Coloring denotes the cell type classification by HALO.
(D) Visual representation of the area calculation of the tumor region. First, a KDE using tumor cells was computed and normalized by the maximum value. Then, all the pixels with a KDE below 0.1 were filtered out (drawn as gray in the middle panel). Lastly, all the remaining pixels were counted, and used for the area estimation.
Abbreviations: *KDE*: kernel density estimation.

Supplementary Figure 2

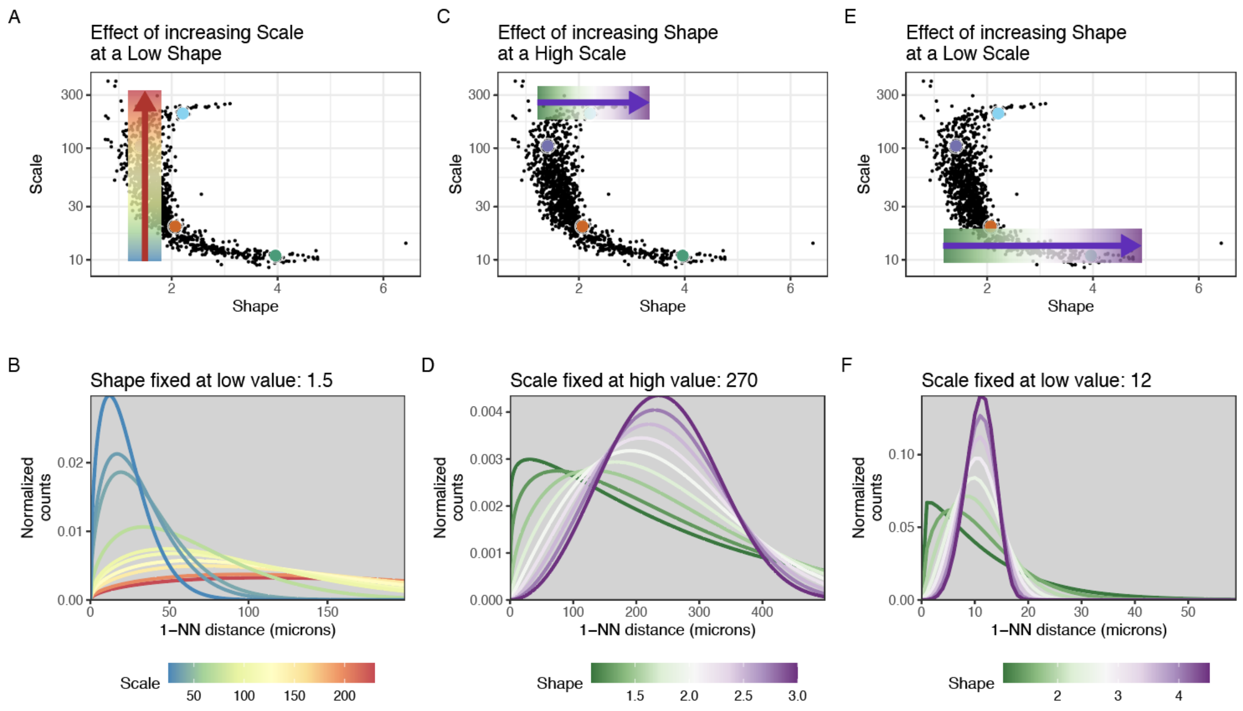

Supplementary Figure 2. Effect of the modeled shape and scale parameters on the Weibull distribution.
A-B) Differences at the Weibull distribution when increasing the scale parameter at a fixed shape.
C-D) Differences at the Weibull distribution when increasing the shape parameter at a fixed scale (high scale).
E-F) Differences at the Weibull distribution when increasing the shape parameter at a fixed scale (low scale).

Supplementary Figure 3

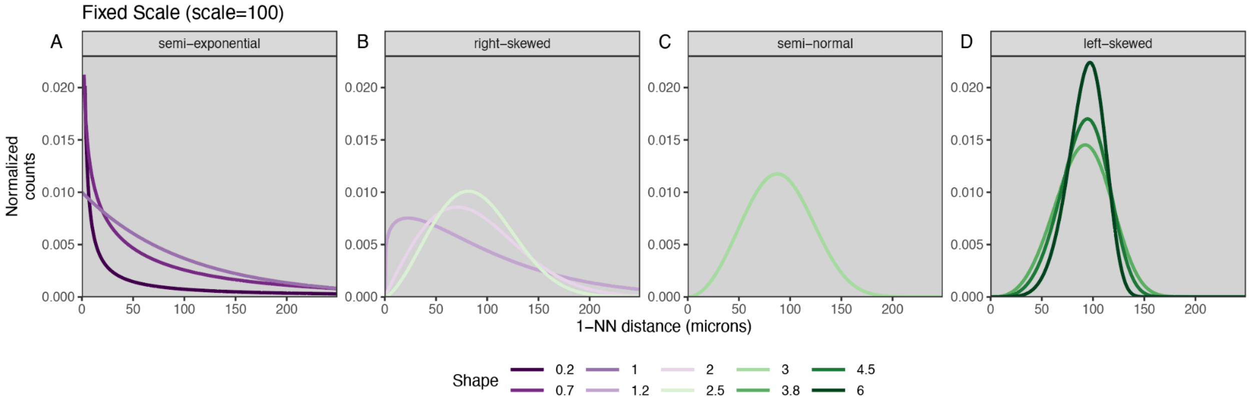

Supplementary Figure 3. Representation of a Weibull distribution when altering the shape parameter at a fixed scale. Each facet groups Weibull distributions grouped by a similar behavior: (A) Represents Weibull distributions resembling a semi-exponential distribution, (B) represents Weibull distributions resembling a right-skewed distribution, (C) represents Weibull distribution resembling a semi-normal distribution and (D) represents Weibull distributions resembling a left-skewed distribution.

Supplementary Figure 4

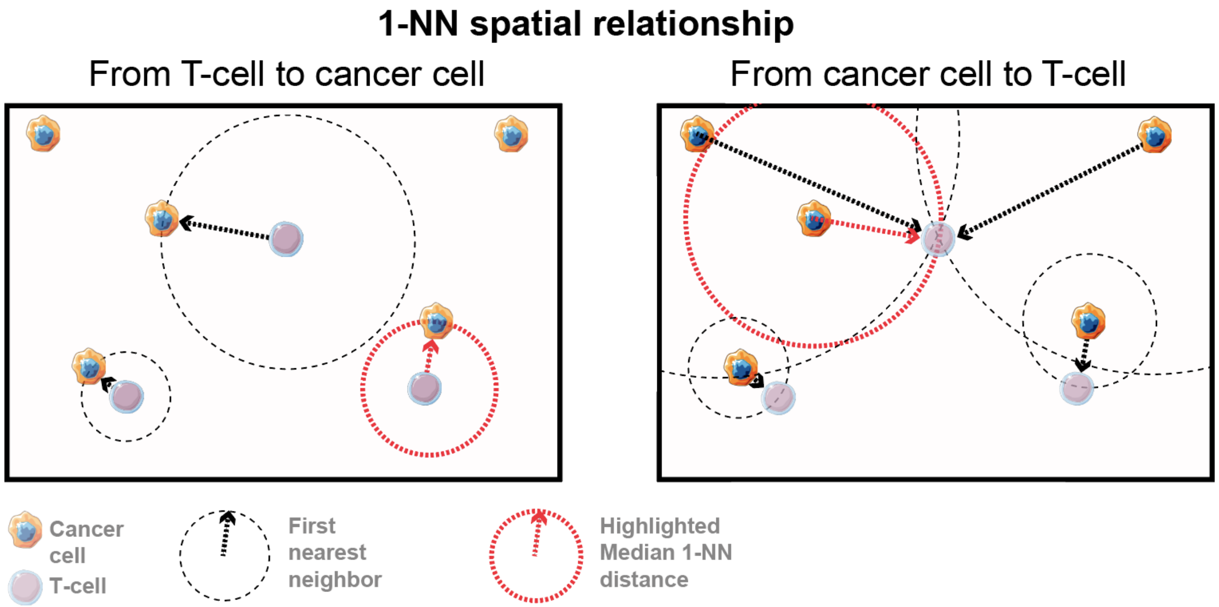

Supplementary Figure 4. SRs were summarized using the 1-NN statistic studied from a reference cell type to a target cell type. The 1-NN statistic is not symmetrical; panels A and B illustrate the statistic when T-cells (left panel) or cancer cells (right panel) are selected as a reference cell type, respectively. Only the 1-NN distances are drawn in each panel. Distances in red indicate the median 1-NN distance for that particular SR.
Icons from panel were adapted from bioIcons (cancerous-cell-1, lymphocytes-4, t-lymphocyte licensed under CC-BY 3.0 Unported by Servier).
Abbreviations: *SR*: spatial relationship; 1-NN: first nearest-neihbor.

Supplementary Figure 5

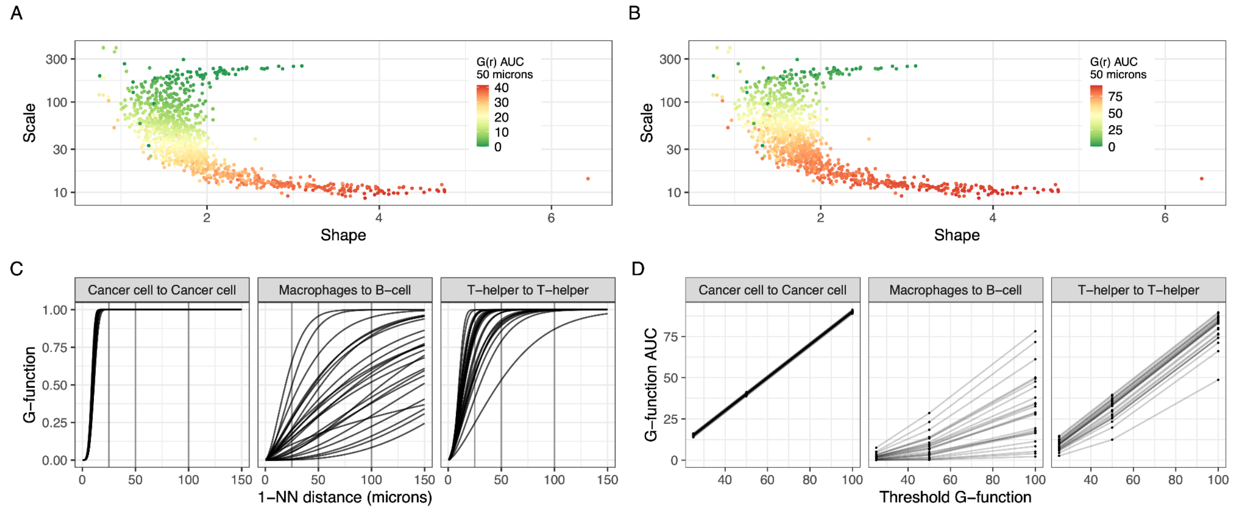

Supplementary Figure 5. Comparison of spatial parameters derived from the 1-NN distance statistic. (A) Scale vs. shape parameter space associated with the G-function evaluated at 12.5 microns (coloring, G-AUC-12).
(B) Scale vs. shape parameter space associated with the G-function evaluated at 50 microns (coloring, G-AUC-50).
(C) G-functions for the B-cell to B-cell, $CD8^{+}$ T-cell to Cancer cell, and Macrophages to B-cell SRs. Lines join samples. Vertical lines denote the thresholds 25, 50 and 100 microns.
(D) G-AUC-T evaluated at 25, 50 and 100 micron threshold for the examples shown in panel (C). Lines join SRs from the same samples.
Abbreviations: *G-AUC-T*: G-function evaluated at a threshold T; *T*: threshold; *AUC*: Area under the curve, *1-NN*: first nearest-neighbor.

Supplementary Figure 6

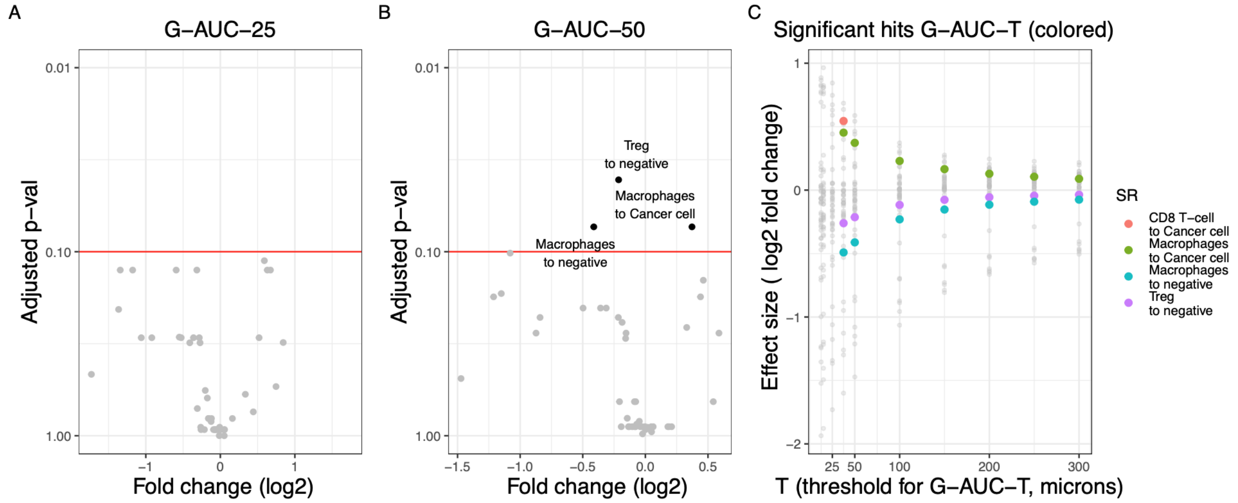

Supplementary Figure 6. **Association of spatial relationships derived from the G-function with response to pre-operative ipilimumab+nivolumab in urothelial cancer.**

(A) Volcano plot showing the fold change on the G-function parameter evaluated at 25 microns (G-AUC-25) between response groups (x-axis, log_2 fold change, n=14 independent responders and n=10 independent non-responders) and statistical significance by Wilcoxon test adjusted by multiple hypothesis testing (y-axis).

(B) Volcano plot showing the fold change on the G-function parameter evaluated at 50 microns (G-AUC-50) between response groups (x-axis, log_2 fold change, n=14 independent responders and n=10 independent non-responders) and statistical significance by Wilcoxon test adjusted by multiple hypothesis testing (y-axis).

(C) Fold change (y-axis, log_2 scale) on the G-function parameter evaluated at different thresholds T (x-axis) between response groups. Fold changes that are significantly associated with response as evaluated with a Wilcoxon test adjusted by multiple hypothesis testing are depicted in color. Non-significant fold changes are depicted in grey. Unless otherwise stated, all statistical tests were two-sided.

Abbreviations: *G-AUC-T*: G-function evaluated at a threshold T; *T*: threshold; *AUC*: Area under the curve; *p-val*: p-value; *SR*: spatial relationship.

Supplementary Figure 7

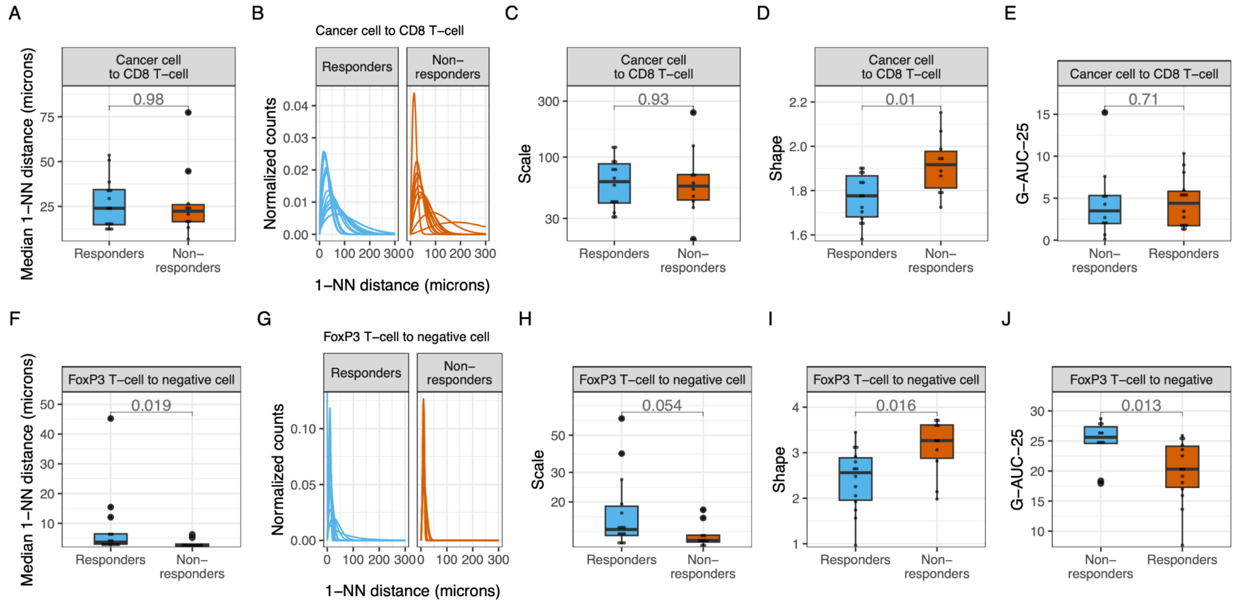

Supplementary Figure 7. Association of spatial relationships with response to pre-operative ipilimumab+nivolumab in urothelial cancer in hits with a weak association.

(A) Median 1-NN distances, (B) associated 1-NN curves, (C) scale parameters and (D) shape parameters and (E) G-AUC-25 parameters stratified by response groups (n=14 independent responders and n=10 independent non-responders) for the SR from cancer cell to $CD8^{+}$ T-cells (top row) and (F) Median 1-NN distances, (G) associated 1-NN curves, (H) scale parameters and (I) shape parameter and (J) G-AUC-25 parameters stratified by response groups for the SR from FoxP3 T-cells to negative cells (bottom row). Statistical significance was assessed by a Mann-Whitney test (panels A, E, F, J) and a t-test (C, D, H, I).

The box plots in each panel show the middle 50% of the data, with the box itself representing the median and the interquartile range (IQR) between the 25th and 75th percentiles. The whiskers extend from the box to the furthest data points within 1.5 times the IQR from the median. Unless otherwise stated, all statistical tests were two-sided and no adjustments for multiple hypotheses were made. Adjusted p-values: FDR_{scale}(cancer cell to $CD8^{+}$ T-cell)=0.98, FDR_{shape}(cancer cell to $CD8^{+}$ T-cell)=0.095, $FDR_{G\text{-}AUC\text{-}25}$(cancer cell to $CD8^{+}$ T-cell)=0.93, FDR_{scale}(FoxP3 T-cell to negative cell)=0.21, FDR_{shape}(FoxP3 T-cell to negative cell)=0.11, $FDR_{G\text{-}AUC\text{-}25}$(FoxP3 T-cell to negative cell)=0.13, $FDR_{G\text{-}AUC\text{-}25}$(FoxP3 T-cell to negative cell)=0.04.

Abbreviations: *1-NN*: first nearest neighbor; *SR:* spatial relationship; *G-AUC-25*: AUC of the G-function evaluated at 25 microns; *AUC*: area under the curve..

Supplementary Figure 8

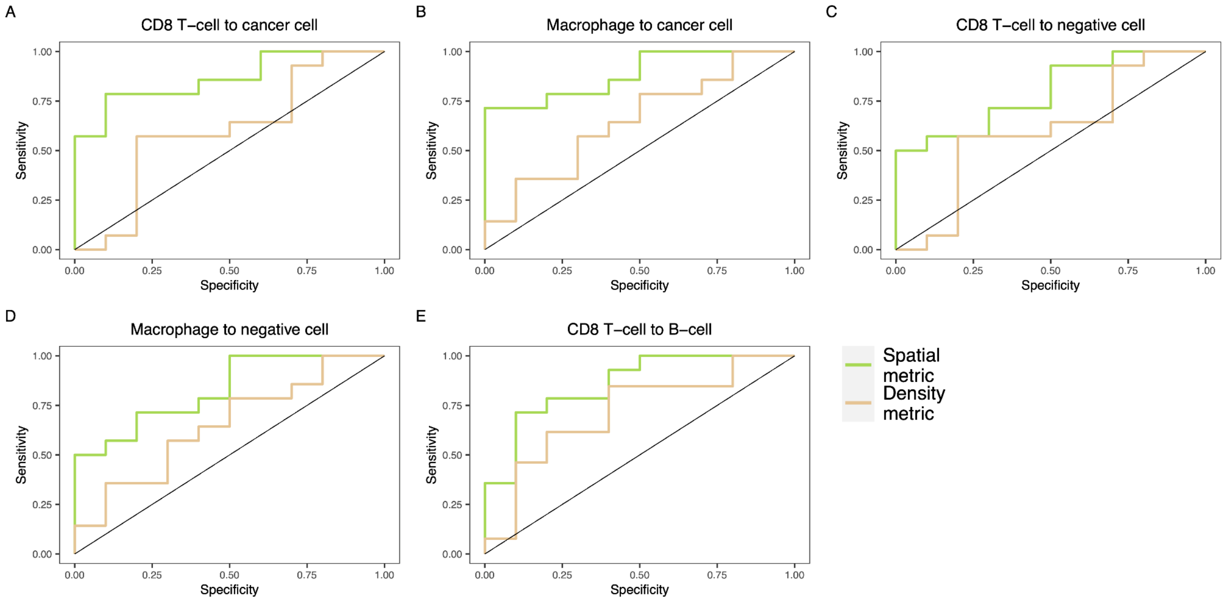

Supplementary Figure 8. **Comparison of the predictive power of associations with response to pre-operative ipilimumab+nivolumab in urothelial cancer between spatial relationships and density parameters.**

ROC curves of the discriminative power of the spatial and density-related parameters involving significant SRs that are associated with response (n=14 independent responders and n=10 independent non-responders), involving (A) CD8 T-cells to cancer cells, (B) Macrophages to cancer cells, (C) CD8 T-cells to negative cells, (D) Macrophages to negative cells, and (E) CD8 T-cells to B-cells. ROC-plots from 'Spatial metrics' (green) were built upon the predictions of a logistic regression model trained on the shape and scale parameters of the associated SR. ROC-plots from 'Density metrics' (orange) were built upon the predictions of a logistic regression model trained on the intratumoral density, stromal density, and exclusion ratio (ratio between stromal and intratumoral density) for the immune cells associated in the SR.

Abbreviations: *SR*: spatial relationship; *ROC*: receiver operating characteristic.

Supplementary Figure 9

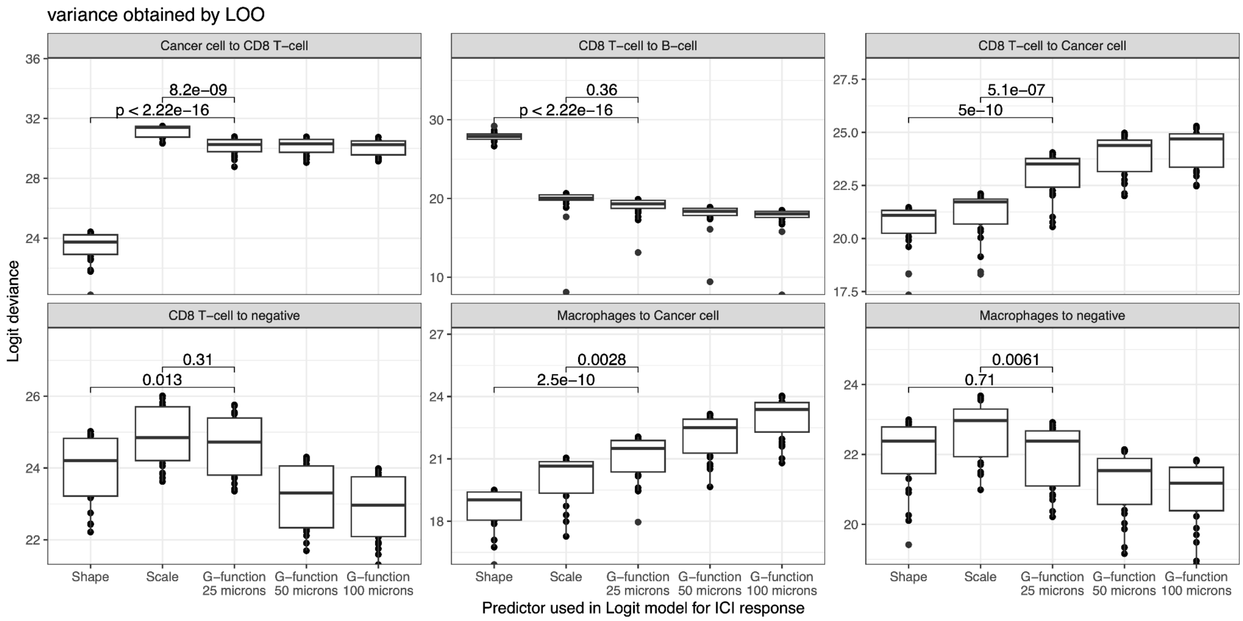

Supplementary Figure 9. **Comparison of predictive models of response to pre-operative ipilimumab+nivolumab in urothelial cancer trained using spatial parameters derived from the 1-NN distance statistic.**

Logistic regression deviance of a univariate logistic regression model predicting ICI response (n=14 independent responders and n=10 independent non-responders) using as a predictor the shape, the scale, or the G-function evaluated at different thresholds (G-AUC-T). Variability on the AIC was evaluated by leave-one-out cross-validation. Each panel denotes models trained using SRs studied for different cell-cell pairwise relationships.

The box plots in each panel show the middle 50% of the data, with the box itself representing the median and the interquartile range (IQR) between the 25th and 75th percentiles. The whiskers extend from the box to the furthest data points within 1.5 times the IQR from the median. Unless otherwise stated, all statistical tests were two-sided. Statistical significance was assessed by a two-sided t-test.

Abbreviations: *SR*: spatial relationship; *ICI*: Immune checkpoint inhibitors; *G-AUC-T*: G-function evaluated at a threshold T; *T*: threshold; logit: logistic regression.

Supplementary Figure 10

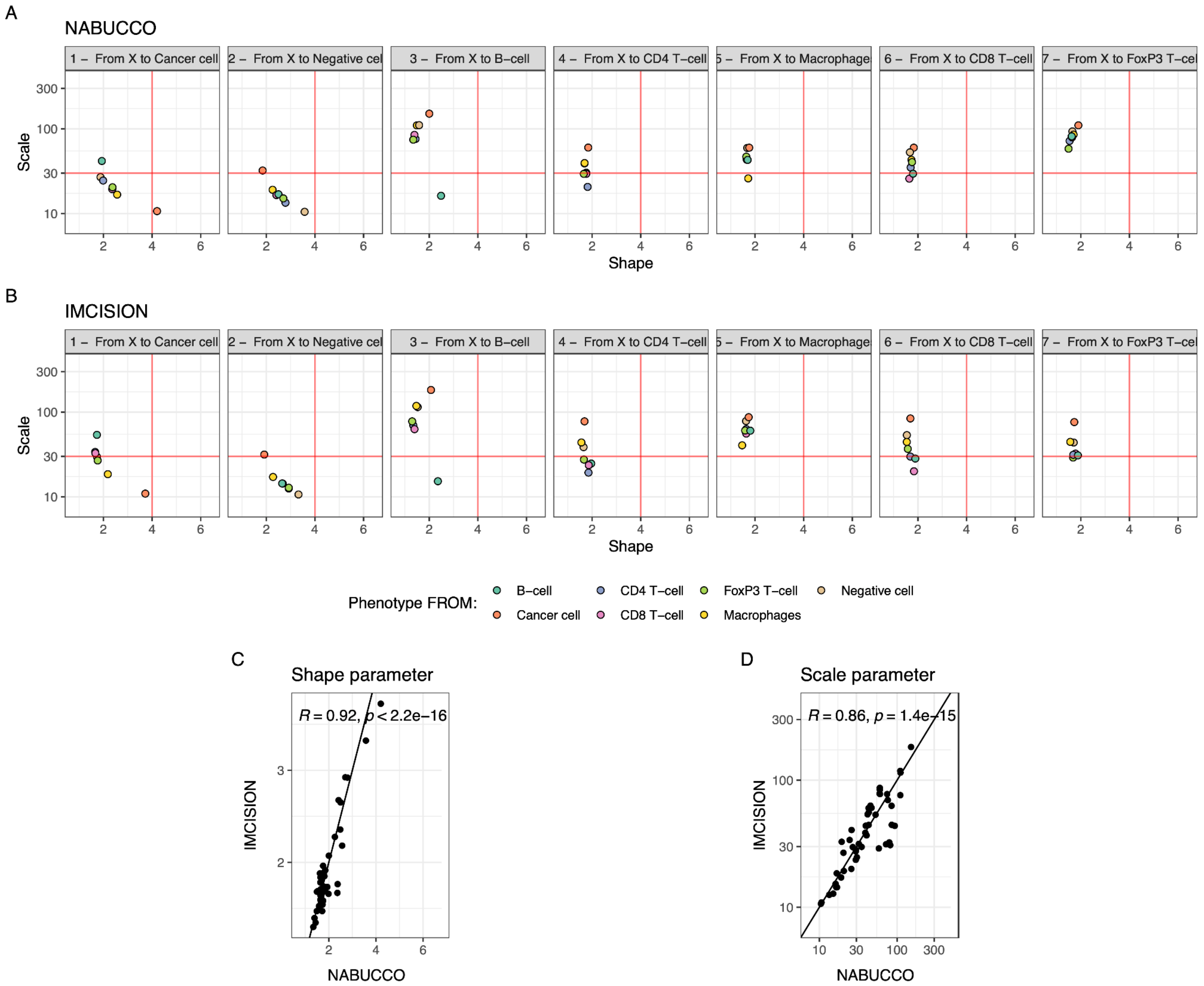

Supplementary Figure 10. Comparison of spatial relationship parameters derived from pre-operative urothelial cancer (NABUCCO) and head and neck cancer (IMCISION) samples.

(A, B) Cohort averages for each SR's cell type pairwise relationships shape and scale parameters in UC (top, NABUCCO, A) and HNSCC (bottom, IMCISION, B). Each facet represents SRs to a specific target cell type. For instance, the first facet represents SRs studied from any reference cell type to cancer cells, and the color indicates the cell type from which the SR was studied (reference cell type).

(C) Correlation between shape parameters quantified in NABUCCO (x-axis) and IMCISION (y-axis). Pearson's coefficient and correlation p-value are shown in the plot.

(D) Correlation between scale parameters quantified in NABUCCO (x-axis) and IMCISION (y-axis). Pearson's coefficient and correlation p-value are shown in the plot.

All statistical tests were two-sided. No adjustments were made to correct for multiple comparisons.

Abbreviations: *SR*: spatial relationship; *UC:* urothelial cancer; *HNSCC*: head and neck squamous cell carcinoma.

Supplementary Figure 11

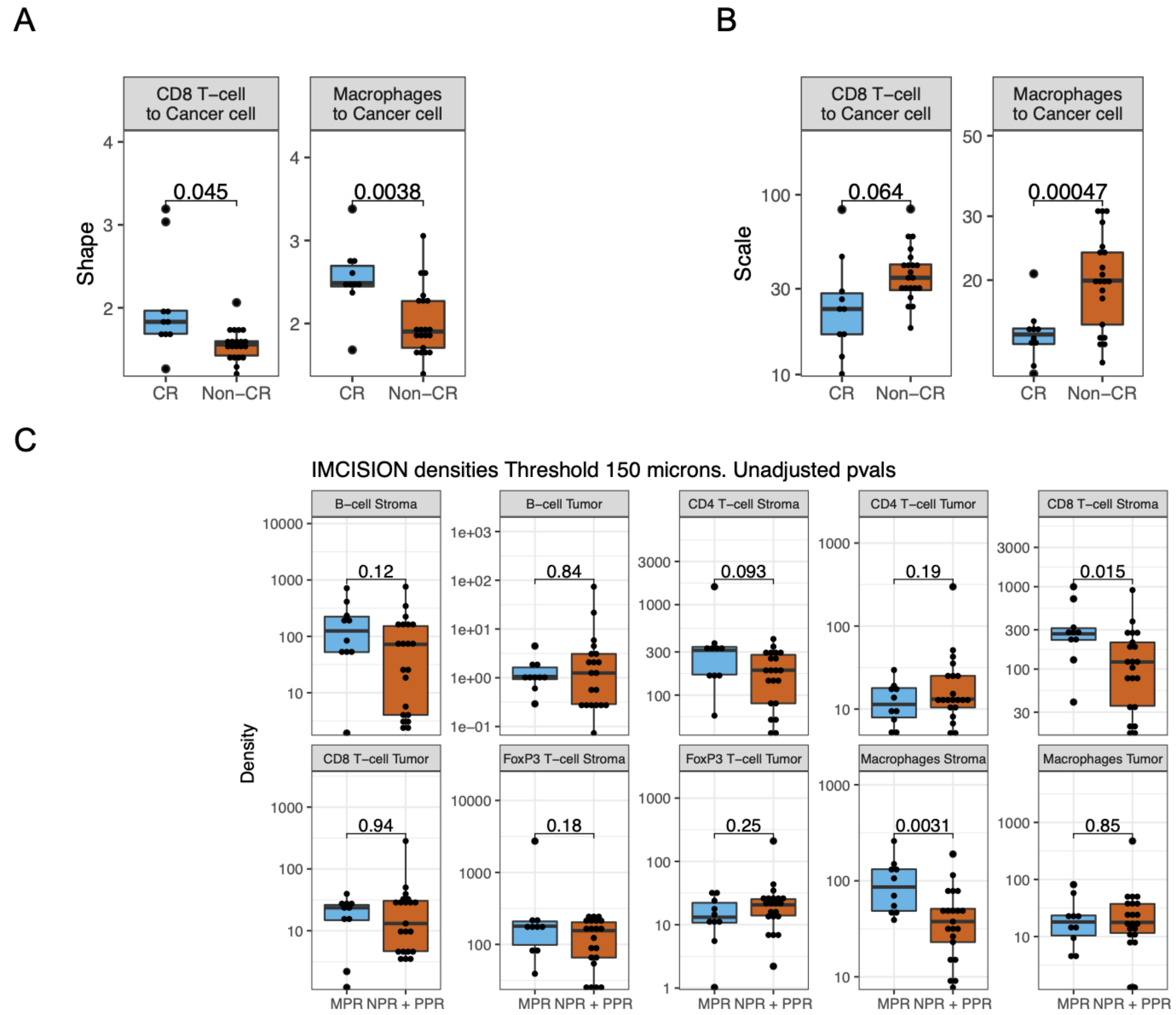

Supplementary Figure 11. **Validation of SR biomarkers of ICI response in head and neck cancer cohort (IMCISION trial).**

(A) Shape parameters for the SRs from $CD8^+$ T-cells to Cancer cells (left) and from Macrophages to cancer cells (right) between ICI response groups. Adjusted p-values: $\text{FDR}^{\text{CD8 T-cell to Cancer cell}}=0.045$, $\text{FDR}^{\text{Macrophages to Cancer cell}}=0.0076$.

(B) Scale parameters for the SRs from $CD8^+$ T-cells to Cancer cells (left) and from Macrophages to cancer cells (right) between ICI response groups. A two-sided t-test was used for comparisons between response groups. Adjusted p-values: $\text{FDR}^{\text{CD8 T -cell to Cancer cell}}=0.064$, $\text{FDR}^{\text{Macrophages to Cancer cell}}=0.00094$.

(C) Immune cell densities between response groups in IMCISION.

The box plots in each panel show the middle 50% of the data, with the box itself representing the median and the interquartile range (IQR) between the 25th and 75th percentiles. The whiskers extend from the box to the furthest data points within 1.5 times the IQR from the median. Unless otherwise stated, all statistical tests were two-sided. A two-sided t-test was used for comparisons between response groups.

Abbreviations: SR: spatial relationship; *ICI*: immune checkpoint inhibitors.

Supplementary Figure 12

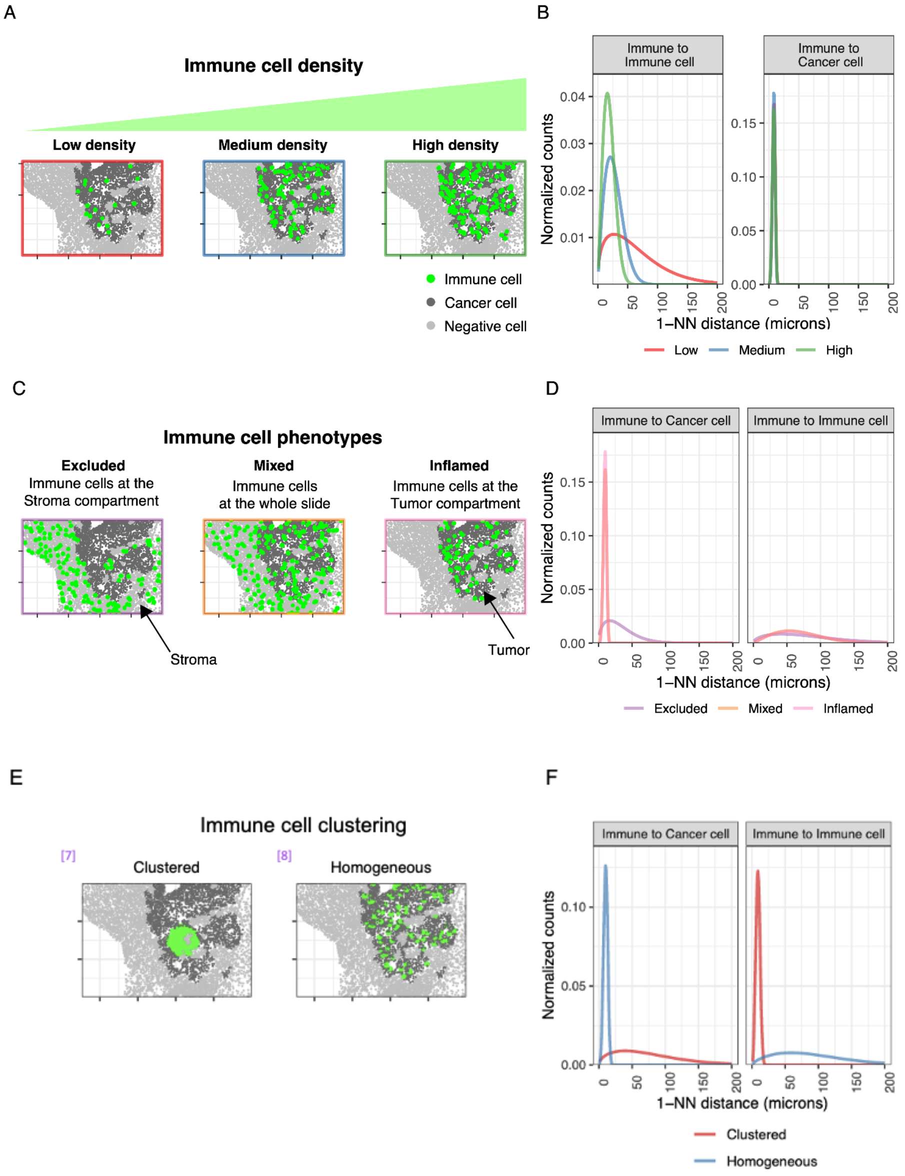

Supplementary Figure 12. Simulation study to quantify sources of variation of the spatial relationship parameters.

Representative data for a sample: (A) Immune cell density was simulated at different values.

(B) 1-NN curves vs immune cell density for three representative SR parameters.

(C) Immune cells were simulated to be present only in the stroma region (*Excluded*, left), both at the stroma and the tumor region (*Mixed*, middle), and only in the tumor region (*Inflamed*, right).

(D) 1-NN curves vs immune cell phenotypes for three representative SR parameters.

(E) Immune cell clustering was simulated with values *Clustered* (left) and *Homogeneous* (right) spatial pattern.

(F) First-nearest neighbor distance curves for the clustered and homogeneous examples from panel C for the SRs studied from Immune to cancer cells and from Immune to immune cells.

Abbreviations: *SR*: spatial relationship; *ICI*: immune checkpoint inhibitors; *1-NN*: first nearest-neighbor.

Supplementary Figure 13

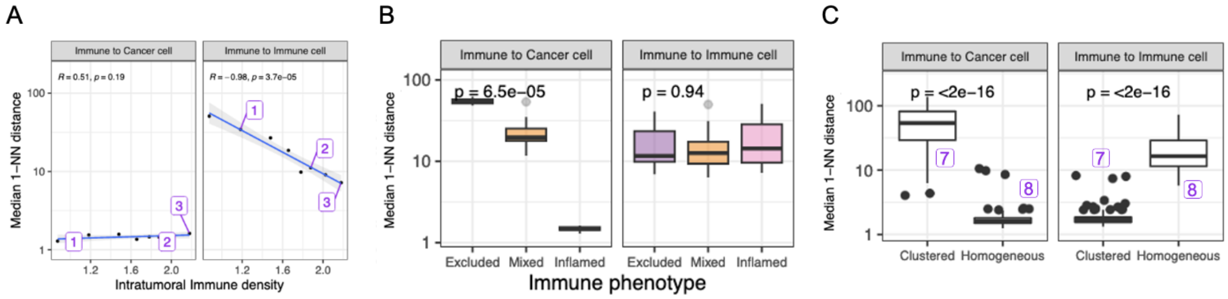

Supplementary Figure 13. Associations between simulation study perturbations and spatial relationship parameters for a representative sample.

(A) Median 1-NN distances vs. simulated intratumoral immune cell density for the SRs from Immune to Cancer cells and from Immune to Immune cell. Two-sided Pearson's moment correlation test was used to test for the association. The Pearson's coefficient and correlation p-value are represented in the plot. 1, 2, and 3 annotations match with Low, Medium and High densities from Supplementary Figure 9A.

(B) Median 1-NN distances vs simulated immune phenotypes for the SRs from Immune to Cancer cells and from Immune to Immune cell. Differences between groups were tested by a Kruskan-Wallis test.

(C) Median 1-NN distances vs simulated immune cell clustering for the SRs from Immune to Cancer cells and from Immune to Immune cell. A two-sided t-test tested differences between groups.

The box plots in each panel show the middle 50% of the data, with the box itself representing the median and the interquartile range (IQR) between the 25th and 75th percentiles. The whiskers extend from the box to the furthest data points within 1.5 times the IQR from the median. All statistical tests were two-sided. Unless otherwise stated, no adjustments for multiple hypothesis testing were made.

Abbreviations: 1-NN: first-nearest neighbor; SR: spatial relationship.

Supplementary Figure 14

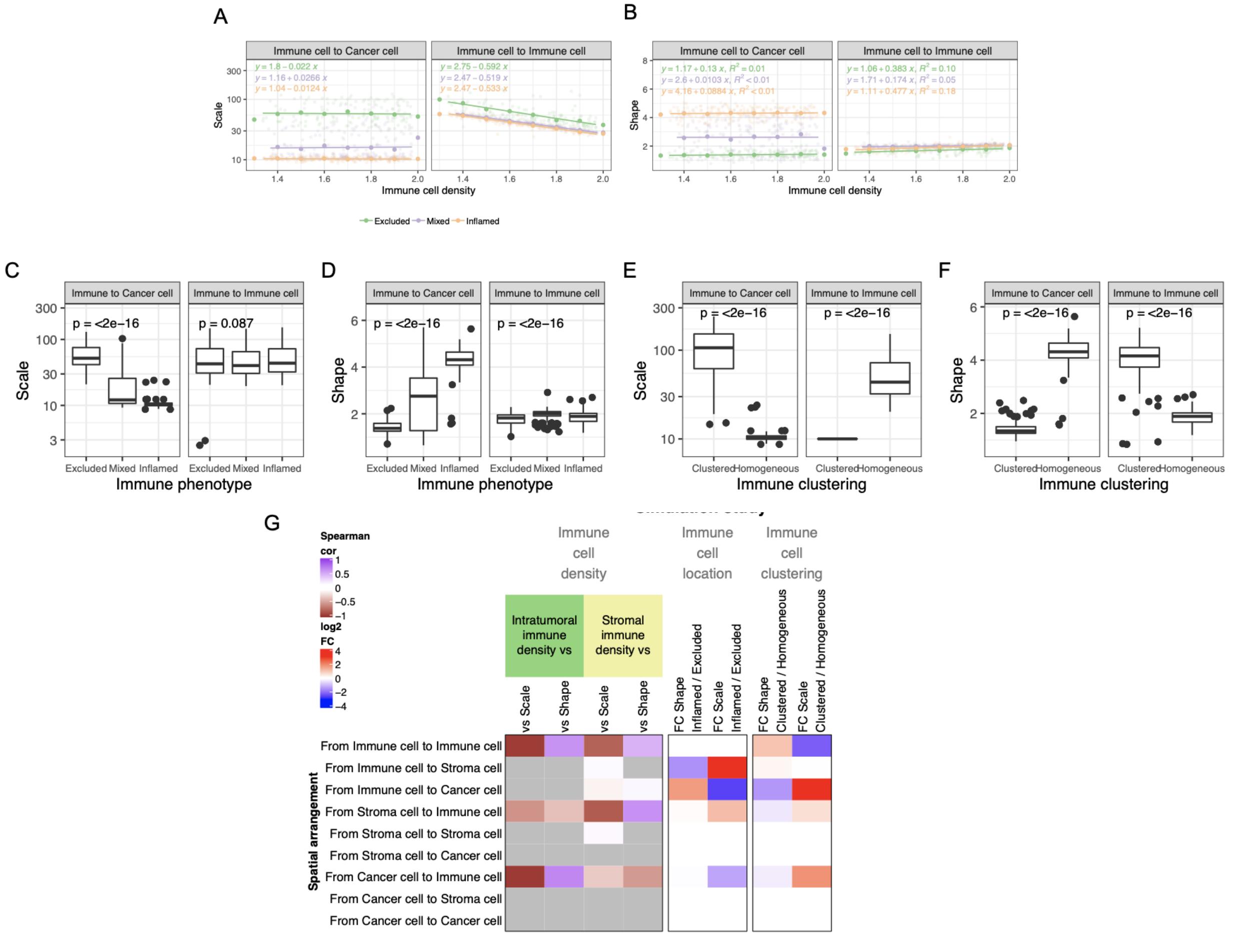

Supplementary Figure 14. Associations between simulation study perturbations and spatial relationship parameters for the whole simulated cohort.

(A) Scatter plot of the scale parameter for two representative SRs for simulations of immune cell at different density values (x-axis) and at distinct tissue compartments, including in the Tumor (Inflamed, orange), Stroma (Excluded, green) and in the Tumor and Stroma (Mixed, purple). A linear model was fitted using the data simulated at each tissue compartment (e.g. Inflamed), and the slope of the fit and significance is highlighted in the figure. Dots represent all simulations' average scale parameters at a discretized density.

(B) Scatter plot of the shape parameter for two representatives SRs for simulations of immune cell at different density values (x-axis) and at distinct tissue compartments, including in the Tumor (Inflamed, orange), Stroma (Excluded, green) and in the Tumor and Stroma (Mixed, purple). A linear model was fitted using the data simulated at each tissue compartment (e.g. Inflamed), and the slope of the fit and significance is highlighted in the figure. Dots represent all simulations' average shape parameters at a discretized density.

(C) Scale parameter distribution for simulations of immune cells present at different tissue compartments (Inflamed: in Tumor; Excluded: in Stroma; Mixed: in Tumor and Stroma). Each boxplot (e.g. Tumor) contains data for different samples and simulations at distinct densities. Statistical association was assessed by an ANOVA test.

(D) Shape parameter distribution for simulations of immune cells present at different tissue compartments (Inflamed: in Tumor; Excluded: in Stroma; Mixed: in Tumor and Stroma). Each boxplot (e.g. Tumor) contains data for different samples and simulations at distinct densities. An ANOVA test assessed statistical association.

(E) Scale parameter distribution for simulations of immune cells following a homogeneous spatial distribution, or a clustered spatial distribution. Each boxplot (e.g. clustered) contains data for different samples and simulations at distinct densities. A t-test assessed statistical association.
(F) Shape parameter distribution for simulations of immune cells following a homogeneous spatial distribution, or a clustered spatial distribution. A t-test assessed statistical association.
(G) First 4 columns: Spearman correlation between the spatial parameters (shape or scale) and intratumoral (green columns) or stromal immune cell density (yellow) for each SR. Fifth and sixth columns: fold change on differences on the shape or the scale parameters between Inflamed and Excluded simulations (non-significant fold changes are coloured in white). Statistical significance was assessed by a t-test. Seventh and eighth columns: fold change on differences on the shape and scale parameters between immune cell clustered or homogeneous simulations by a t-test. Non-significant correlations or associations are labeled in gray and white.
The box plots in each panel show the middle 50% of the data, with the box itself representing the median and the interquartile range (IQR) between the 25th and 75th percentiles. The whiskers extend from the box to the furthest data points within 1.5 times the IQR from the median. All statistical tests were two-sided. Unless otherwise stated, no adjustments for multiple hypothesis testing were made.
Abbreviations: *UC*: urothelial cancer; *SR:* Spatial relationship.

Supplementary Figure 15

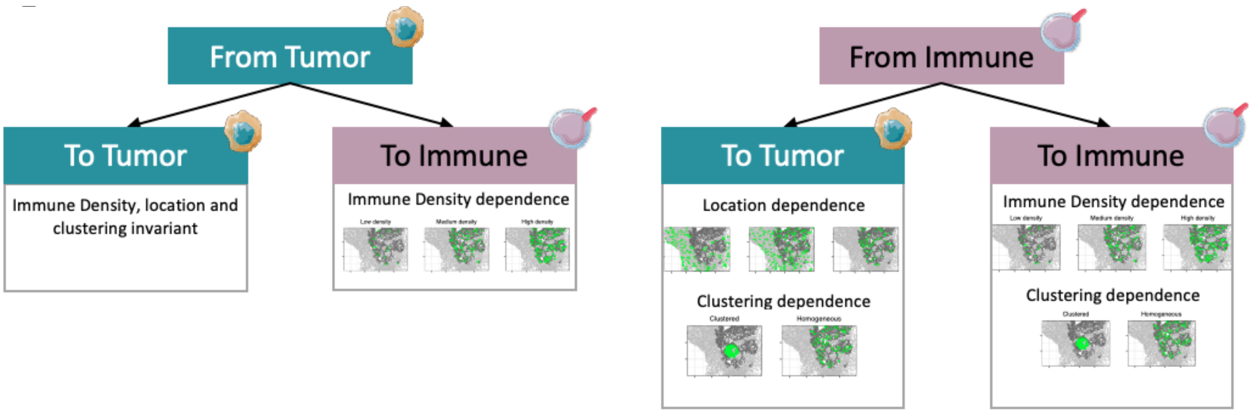

Supplementary Figure 4. Interpretations on the associations between simulation study perturbations and spatial relationship parameters for the whole simulated cohort.

Summary of associations between simulated perturbations and effect on SR parameters by reference and target cell type by aggregating data for the whole cohort (Supplementary Figure 21H). Here, the interpretation of when *Cancer cells* are replaced by *Negative cells* are analogous (abundant cell types). Immune cells are referenced in text text as rare cell types.

Icons from panel adapted from bioIcons (cancerous-cell-1, lymphocytes-4, t-lymphocyte licensed under CC-BY 3.0 Unported by Servier).

Abbreviations: *SR*: spatial relationship.

Supplementary Notes

Supplementary Note 1: Quantifying sources of variation of the spatial relationship parameters

To investigate whether SR metrics are susceptible to changes in immune cell density, we performed a simulation study based on the real patient mIF data (Supplementary Methods, *Spatial analysis: a simulation study to quantify spatial relationship parameters sources of variation*). We preserved the spatial positions of cells (but not their identity) and the tumor and stroma delineation. We then placed immune cells at an increasing density at the preserved cell positions for the cohort. Supplementary Figure 12A shows a representative sample where the immune cells are only placed in the cancer compartment, i.e., replacing a cancer cell with an immune cell. We varied the percentage of immune cells in the tumor compartment from 1 to 10%, where the percentage is a fraction of the total number of cells on a slide. This range is representative of the variation in immune cell fraction we observed in our cohort (2-1583 immune cells/micron^2).

We started by simulating multiple intratumoral immune cell densities, which included *Low* (1% of immune cells), *Medium* (4% of immune cells), or *High* (7% of immune cells) densities (Supplementary Figure 12A). We then estimated all the pairwise SR parameters between cancer, immune and negative cells. As expected, an increase in immune cell density decreased the 1-NN distances between immune cells (Supplementary Figure 12B, and Supplementary Figure 13A, right panel). The associated medians for each 1-NN spatial distribution shown in Supplementary Figure 12B confirmed these findings (Supplementary Figure 13A). In contrast, changes in immune cell density did not affect the 1-NN curves nor the metrics quantifying the SR from immune to cancer cells (Supplementary Figure 13A). Our findings showed little variation between the different patient samples (Supplementary Figures 14A-B). In summary, we found that density can affect the SR metrics, but that is highly dependent on the cell types involved and the directionality of the relationship.

We can conceive the following immune cells spatially distributions giving rise to the following configurations of immune phenotypes[30]. First, being Excluded (higher immune cell abundance in the stroma), Inflamed (high immune cell abundance in the tumor), and Desert (low immune cell abundance in the tumor and stroma). In order to quantify the effect of immune phenotypes on the SR metrics, we simulated the associated extreme scenarios of the immune phenotypes, and named them as *Excluded* (immune cells only in the stroma and not in the tumor), *Mixed* (immune cells both in the stroma and tumor), and *Inflamed* (immune cells only in the tumor), all at distinct immune cell densities (Supplementary Figure 12C). As expected, the different immune phenotypes have a distinct effect on the SR of immune to cancer cells. In contrast, the SR immune to immune cells remains unaffected, similar to the observation when the immune cell density was varied (Supplementary Figures 12D, 13B). Notably, these effects were remarkably stable across the complete cohort (small variance in Supplementary Figure 14A-B),

indicating that the specific positions of cells and the arrangement of tumor and stroma in a particular tumor do not have a strong effect on the simulated SRs. Cases from a particular configuration (e.g., *Inflamed*) consisted of aggregated simulations of distinct immune cell densities compatible with such configuration to assess the true effect between immune phenotypes in our comparisons (e.g., *Inflamed* vs. *Excluded*). Again, little sample variability within the whole cohort was observed between the associations of immune phenotype perturbation and SR parameters (Supplementary Figure 14C-D).

Next, we compared the SR metrics for homogeneously and heterogeneously distributed immune cells. Examples of such instances include T-cells homogeneously infiltrating a tumor or B-cells forming immune cell clusters resulting in non-homogeneous distributions of B-cells. We simulated these two scenarios (Supplementary Figure 12E) and compared the associated SR metrics. We found that immune cell clustering can affect the SR metrics from immune to immune cells and from immune to cancer cells (Supplementary Figures 12F, 13C, 14E-F).

To summarize our findings, we aggregated simulations for all the cell type pairwise relationships and samples (Supplementary Figure 14G). Changes in the SR parameters upon the posed perturbations highly depend on which reference and target cell types were studied (Supplementary Figure 15). The SRs between abundant cell types (i.e., cancer and negative cells) were not altered upon such perturbations. In contrast, the SRs between rare cell types (i.e., immune cells) were affected by the density or local clustering perturbations and SRs from abundant to immune cells were affected by the density perturbations. Moreover, immune phenotypes and clustering perturbations affected SRs from immune to cancer cells. In conclusion, we identified multiple factors that affect the magnitude of SR parameters, which depending on the cell type context being studied, need to be considered in downstream analysis.

Supplementary Figures

Supplementary Methods

Spatial analysis: a simulation study to quantify spatial relationship parameters sources of variation

Using the segmentation between the tumor and stroma compartments and the tissue architecture, we simulated different states of immune cell infiltration or cell location to quantify sources of variation in the spatial patterns. We used our original samples' point patterns and the delineated tumor and stroma compartments in all the simulations. We altered the abundance and location of three cell types: immune, cancer, and stroma.

First, we simulated immune cell density at different values. Here, all the cells belonging to the tumor compartment were labeled as cancer cells, and the cells belonging to the stroma compartment were labeled as stroma cells. Then, we randomly re-labeled cells a fraction of cancer cells (at a 1%, 2%, 5%, 7%, and 10% fraction) as immune cells, in which the SR of the immune cells resembled a homogeneous spatial distribution. Analogously, the same simulation study was carried out by randomly re-labeling stroma cells or altogether re-labeling cancer and stroma cells. These perturbations led to three different simulation studies (immune cells only in tumor, only in stroma, or in tumor and stroma), in which the SR parameters from/to cancer, immune and stromal cells (3x3=9 SRs) were estimated. For each simulation, the immune cell density (either intratumoral or stromal) was associated with the distinct SR Weibull parameters (shape and scale) obtained for the 9 SRs being studied.

In a second simulation study, we compared the SR parameters between the previous three different simulations that matched distinct immune phenotypes, being *Inflamed* (simulated immune cells in Tumor), *Excluded* (simulated immune cells in Stroma), and *Mixed* (simulated immune cells altogether in Tumor and Stroma). This study aggregated and compared simulations between groups, e.g., *Inflamed* vs *Excluded (*i.e., *Inflamed* combines simulations with an inflamed phenotype at multiple densities), as we aimed to quantify the global effect of the perturbation.

Lastly, we simulated differences in the local immune cell arrangement by perturbing the clustering of immune cells. In *Clustered* simulations, we allocated the immune cells next to each other by setting an anchor point in which immune cells were present at different abundances (1%, 2%, 5%, and 10% fraction of cells being immune cells). Using this simulation (*Clustered*), we compared the SR parameters with the previous simulation, in which immune cells were placed along the tissue following a *Homogeneous* spatial distribution.
