# Supplementary material for: Spatial relationships in the urothelial and head and neck tumor microenvironment predict response to combination immune checkpoint inhibitors
Source: Nat Commun. 2024 Mar 21;15:2538. doi: 10.1038/s41467-024-46450-1 (PMC10957922; doi:10.1038/s41467-024-46450-1)
Supplement: Supplementary file 3 — Description of Additional Supplementary Files [file 41467_2024_46450_MOESM3_ESM.pdf]

Description of Additional Supplementary Files

Supplementary Data 1. Multiplex immunofluorescence derived data (spatial parameters and densities).

Sheet 1: Density parameters and exclusion ratios quantified from the NABUCCO cohort. Abbreviations: CR: Responder; NCR: Non-responder.

Sheet 2: Spatial relationship parameters quantified from the NABUCCO cohort (shape, scale, and G-function evaluated at 25, 50 and 100 microns). Abbreviations: CR: Responder; NCR: Non-responder; AUC: area under the curve; SR: spatial relationship.

Sheet 3: Spatial relationship parameters quantified from the IMCISION cohort. Abbreviations: CR: Responder; NCR: Non-responder; SR: spatial relationship.
