# Supplementary material for: Spatial relationships in the urothelial and head and neck tumor microenvironment predict response to combination immune checkpoint inhibitors
Source: Nat Commun. 2024 Mar 21;15:2538. doi: 10.1038/s41467-024-46450-1 (PMC10957922; doi:10.1038/s41467-024-46450-1)
Supplement: Supplementary file 5 — Reporting Summary [file 41467_2024_46450_MOESM5_ESM.pdf]

nature portfolio

Corresponding author(s): Alberto Gil-Jimenez

Last updated by author(s): Feb 2, 2024

Reporting Summary

Nature Portfolio wishes to improve the reproducibility of the work that we publish. This form provides structure for consistency and transparency in reporting. For further information on Nature Portfolio policies, see our Editorial Policies and the Editorial Policy Checklist.

Statistics

For all statistical analyses, confirm that the following items are present in the figure legend, table legend, main text, or Methods section.

n/a	Confirmed	
☐	☒	The exact sample size (*n*) for each experimental group/condition, given as a discrete number and unit of measurement
☐	☒	A statement on whether measurements were taken from distinct samples or whether the same sample was measured repeatedly
☐	☒	The statistical test(s) used AND whether they are one- or two-sided<br>*Only common tests should be described solely by name; describe more complex techniques in the Methods section.*
☐	☒	A description of all covariates tested
☐	☒	A description of any assumptions or corrections, such as tests of normality and adjustment for multiple comparisons
☐	☒	A full description of the statistical parameters including central tendency (e.g. means) or other basic estimates (e.g. regression coefficient) AND variation (e.g. standard deviation) or associated estimates of uncertainty (e.g. confidence intervals)
☐	☒	For null hypothesis testing, the test statistic (e.g. *F*, *t*, *r*) with confidence intervals, effect sizes, degrees of freedom and *P* value noted<br>*Give P values as exact values whenever suitable.*
☒	☐	For Bayesian analysis, information on the choice of priors and Markov chain Monte Carlo settings
☒	☐	For hierarchical and complex designs, identification of the appropriate level for tests and full reporting of outcomes
☐	☒	Estimates of effect sizes (e.g. Cohen's *d*, Pearson's *r*), indicating how they were calculated

Our web collection on statistics for biologists contains articles on many of the points above.

Software and code

Policy information about availability of computer code

Data collection: Multiplex immunofluorescence-derived phenotype and marker spatial locations were collected in .tsv files using HALO (Indica Labs, V2.3).

Data analysis: R 3.6.3 with the following packages:
* Spatstat 1.64
● Dplyr 1.0.4
● Fitdistrplus 1.1.3
● Patchwork 1.1.1
● Survival 1.3.24
● ComplexHeatmap 2.2
● Circlize 0.4.12
● Glmnet 4.1.1
● RColorBrewer 1.1.2
● Nlme 3.1.144
● Spastat 1.7.0
● Ggpubr 0.4.0
● Ggrepel 0.9.1
● Plyr 1.8.6
● Tidyverse 1.3.0
● Ggplot 2.3.3.3

March 2021

● Tibble 3.0.6
● ggrastr version 1.0.1
● pROC 1.17.0.1

The code used for this manuscript is deposited on a Github repository: https://github.com/tropicalberto/nabucco_spatial_manuscript

For manuscripts utilizing custom algorithms or software that are central to the research but not yet described in published literature, software must be made available to editors and reviewers. We strongly encourage code deposition in a community repository (e.g. GitHub). See the Nature Portfolio guidelines for submitting code & software for further information.

Data

Policy information about availability of data

All manuscripts must include a data availability statement. This statement should provide the following information, where applicable:

- Accession codes, unique identifiers, or web links for publicly available datasets
- A description of any restrictions on data availability
- For clinical datasets or third party data, please ensure that the statement adheres to our policy

Multiplex immunofluorescence primary data (immune cell type and spatial coordinates) used for this manuscript will be made available upon reasonable academic request and within the limitations of the provided informed consent by the corresponding author upon reasonable request upon acceptance. The institutional review board of the Netherlands Cancer Institute will review each request. After approval, the researcher must sign the Netherlands Cancer Institute data access agreement.
Multiplex immunofluorescence derived data (spatial parameters) is made available as supplementary information (supplementary tables).

Human research participants

Policy information about studies involving human research participants and Sex and Gender in Research.

Reporting on sex and gender: Sex or gender data were not considered in the study design of this manuscript.

Population characteristics:

Clinical trial, treatment and sample characteristics for the cohorts used in this study are published in Table 1 from the manuscript.

Populations characteristics from the cohorts are published in the clinical data original manuscripts: https://doi.org/10.1038/s41591-020-1085-z and https://doi.org/10.1038/s41467-021-26472-9

Population characteristics NABUCCO: Adult patients, male or female, with high-risk resectable urothelial cancer (upper urinary tract allowed), defined as:
- cT3-4aN0M0 OR cT1-4aN1-3M0
Patients refused neoadjuvant cisplatin based chemotherapy or in whom neoadjuvant cisplatin based therapy is not appropriate,
WHO performance status 0-1, naïve for CTLA-4/PD-1/PD-L1 immunotherapy, and more than 18 years old.

Population characteristics IMCISION: Thirty-two patients were enrolled. Eligible patients, male or female, were 18 years of age or older and had T2–T4, N0–N3, M0 head and neck squamous cell carcinoma (HNSCC) and an indication for curatively intended extensive head and neck surgery. Patients with recurrent or residual HNSCC were eligible as long as they had an indication for salvage surgery with curative intent. All patients had a World Health Organization Performance Status of 0 or 1 and adequate end organ function. Main exclusion criteria were the presence of autoimmune disease, human immunodeficiency virus or hepatitis B/C infection; prior immunotherapy targeting CTLA-4, PD-1, or PD-L1 and use of immunosuppressive medication.

Recruitment: Recruitment characteristics from the cohorts are published in the clinical data original manuscripts https://doi.org/10.1038/s41591-020-1085-z and https://doi.org/10.1038/s41467-021-26472-9

Ethics oversight: The Netherlands Cancer Institute approved the study protocols as specified in the clinical data original manuscripts: https://doi.org/10.1038/s41591-020-1085-z and https://doi.org/10.1038/s41467-021-26472-9

Note that full information on the approval of the study protocol must also be provided in the manuscript.

Field-specific reporting

Please select the one below that is the best fit for your research. If you are not sure, read the appropriate sections before making your selection.

[x] Life sciences   [ ] Behavioural & social sciences   [ ] Ecological, evolutionary & environmental sciences

For a reference copy of the document with all sections, see nature.com/documents/nr-reporting-summary-flat.pdf

Life sciences study design

All studies must disclose on these points even when the disclosure is negative.

Sample size	The datasets used in this manuscript were collected from two clinical trials: NABUCCO and IMCISION.<br><br>NABUCCO sample size:<br>The primary endpoint of this trial was feasibility, testing whether preoperative ipilimumab and nivolumab is feasible within 12 weeks from first infusion and does not delay surgical resection, as this is an endpoint that is clinically meaningful for this population. We set the desired resection rate by 12 weeks at 90% of patients. The lower statistical boundary for futility was set at 60%. For 24 patients and one-sided alpha=0.025, the power of rejecting 60% resection rate, under the alternative of 90%, is 91%. Treatment efficacy (pCR) was selected as a secondary endpoint, and defined by the percentage of pathological complete response (complete absence of neoplastic cells, pT0N0) at surgical resection. For the efficacy endpoint we considered the disease burden of this cohort, which is higher than a typical neoadjuvant cohort, and cisplatin-ineligibility/refusal. We determined that a 40% pCR rate would be desirable, while 14% or lower would clearly not warrant further investigation of treatment. Under those assumptions, 24 evaluable patients provide 90% power to detect treatment efficacy with the one-sided alpha of 0.05. At least 7 responders are required.<br><br>IMCISION sample size:<br>Safety and feasibility, defined as no delay in surgery due to immune-related adverse events beyond week 6, were the primary endpoints of the phase Ib study. Twelve patients were included in phase Ib in a double 3+3 design. The first 3 patients were treated with nivolumab monotherapy prior to surgery. When safety according to primary outcome was established in all 3 patients, 3 additional patients were treated with neoadjuvant nivolumab monotherapy. If the primary endpoint was met in at least 5 of 6 total nivolumab monotherapy patients, 3 new patients were included and treated with neoadjuvant nivolumab with ipilimumab. After again establishing safety of preoperative nivolumab + ipilimumab according to the primary endpoint in these 3 patients, 3 additional patients were included and treated with neoadjuvant nivolumab + ipilimumab. If the primary endpoint was met in at least 5 of 6 nivolumab + ipilimumab patients, an exploratory phase IIa expansion cohort would be opened in which 20 additional patients would be treated with the most intense regimen (i.e. nivolumab + ipilimumab), if proven tolerable in phase Ib.<br><br>For phase Ib, no formal sample size calculation was performed and a 3+3 design was used. For the sample size calculation of phase IIa, a 33% incidence of pathological response was hypothesized. A pathological response rate less than 10% would be considered clinically irrelevant. Combining phase Ib and IIa would yield a minimum of 26 patients treated with either nivolumab monotherapy or nivolumab + ipilimumab. Assuming a pathological response in 33% of 26 patients would allow for the rejection of an actual pathological response rate of < 10% in the population at large with 95% power and 95% (one-sided) confidence.
Data exclusions	No data was excluded for the manuscript (all samples)<br>In the IMCISION trial, data from one sample was not profiled in multiplex immunofluorescecne and therefore not analyzed.<br>In the IMCISION trial dataset, data from only one arm (ipilimumab+nivolumab arm) was used in this study
Replication	Code revisions and figures were thoroughly checked.<br>Replication was not done on the samples level
Randomization	The datasets used in this manuscript were collected from two clinical trials: NABUCCO and IMCISION.<br><br>Randomization NABUCCO:<br>There was no randomization. All participant were checked for eligibility using the eligibility criteria in the protocol. No waivers for eligibility criteria were given, no protocol violations were observed by the study monitor.<br>Covariates were controlled by using clearly defined inclusion and exclusion criteria with patient characteristics presented in Fig. 1. In addition, the primary endpoint (feasibility) and secundary efficacy endpoint involved objective outcome measures (surgery within 12 weeks, pCR rate), limiting the impact of potential baseline covariates. The inclusion of stage III patients with extensive/bulky disease improved the assessment and reliability of pathological outcome measures.<br><br>Randomization IMCISION:<br>There was no randomization. Phase Ib patients were treated with nivolumab (first n=6) or nivolumab + ipilimumab (next n=6) based on order of accrual. All phase IIa patients would be treated with the same dosage of nivolumab + ipilimumab, after this regimen proved safe and feasible in the phase Ib.<br>Covariates were controlled by using clearly defined inclusion and exclusion criteria as summarized in the manuscript, and fully detailed in the Study Protocol.
Blinding	The datasets used in this manuscript were collected from two clinical trials: NABUCCO and IMCISION.<br><br>Blinding NABUCCO:<br>Blinding was not done. Patients and treating physicians were aware of the therapeutic intervention, as this involved a single-arm study with only one treatment option<br><br>Blinding IMCISION:<br>IMCISION was an open-label study. The neoadjuvant treatment given in phase Ib was pre-determined and based on order of accrual as described under 'Sample Size'. As all phase IIa patients would be treated with the most intense regimen (nivolumab + ipilimumab) after its feasibility had been established in phase Ib, treating physicians and investigators would need to be aware of the neoadjuvant treatment in phase Ib; blinding was therefore not performed. In phase IIa, only one neoadjuvant treatment regimen would be administered to all, making

blinding senseless.

Reporting for specific materials, systems and methods

We require information from authors about some types of materials, experimental systems and methods used in many studies. Here, indicate whether each material, system or method listed is relevant to your study. If you are not sure if a list item applies to your research, read the appropriate section before selecting a response.

Materials & experimental systems

n/a	Involved in the study
☐	☒ Antibodies
☒	☐ Eukaryotic cell lines
☒	☐ Palaeontology and archaeology
☒	☐ Animals and other organisms
☐	☒ Clinical data
☒	☐ Dual use research of concern

Methods

n/a	Involved in the study
☒	☐ ChIP-seq
☒	☐ Flow cytometry
☒	☐ MRI-based neuroimaging

Antibodies

Antibodies used

NABUCCO antibodies:
CD3: 1/400 dilution, Clone P7, Cat RM-9107-S, ThermoScientific
CD8: 1/100 dilution, Clone C8/144B, Cat M7103, DAKO
CD68: 1/500 dilution, Clone KP1, M0814, Dako
FoxP3: 1/50 dilution, Clone 236A/47, Cat ab20034, Abcam
CD20: 1/500 dilution, Clone L26, cat M0755, Dako
PanCK: 1/100 dilution, Clone AE1AE3, Cat MS-343P, Thermo Scientific

IMCISION antibodies:
IMCISION antibodies
- CD3, clone SP7, ThermoScientific, CatalogNo: RM-9107-S, LotNo: 9107S1805A
- CD8, clone C8/144B, DAKO / Agilent, CatalogNo: M7103, LotNo: 20048132
- CD68, clone KP1, DAKO / Agilent, CatalogNo: M0814, LotNo: 20040389
- FoxP3, clone 236A/47, DAKO / Agilent, CatalogNo: ab20034, LotNo: GR3220121-1
- CD20, clone L26, DAKO / Agilent, CatalogNo: M0755, LotNo: 20038880
- PanCK, clone AE1AE3, Thermoscientific, CatalogNo: MS-343P, LotNo: 343P1205H

Validation

NABUCCO antibodies:
Validation of all antibodies for the use in brightfield immunohistochemistry staining was previously done following certified diagnostic laboratory protocols. Then, the same antibodies were used with the OPAL immunofluorescence (IF) dyes.
Tests to assess 1) the best position within the multiplex sequence, 2) the best concentration and 3) the best incubation times were performed for each individual antibody.
Initially a set-up on lymphoid tissue was done, then Tissue micro arrays (TMA) with 50 (3 cores 0.6 mm per tumor tissue) bladder tumor tissues were stained and evaluated with a pathologist to evaluate the specificity of the multiplex staining.

IMCISION antibodies:
Each antibody staining protocol has been developed and validated in diagnostic setting under standard operating procedures in a certified pathology lab (EN ISO15189, M258).
Each new antibody lot is validated by testing multiple dilutions and evaluating them with the pathologist in a standardized method, using positive control tissues suitable for the antibody (images and protocol details available upon request).

Clinical data

Policy information about clinical studies

All manuscripts should comply with the ICMJEguidelines for publication of clinical research and a completedCONSORT checklist must be included with all submissions.

Clinical trial registration	ClinicalTrials.gov: NCT03387761 (NABUCCO) and NCT03003637 (IMCISION)
Study protocol	Study protocols are attached as appendix in the clinical data original manuscripts: https://doi.org/10.1038/s41591-020-1085-z and https://doi.org/10.1038/s41467-021-26472-9
Data collection	Data collection NABUCCO:<br>Patients were enrolled at the Netherlands Cancer Institute between February 2018 and February 2019.<br>The data cut-off in the current manuscript was 1st September 2019.<br>Clinical data was collected through an eCRF by the clinical trial department of the Netherlands Cancer Institute. Clinical data was analyzed by the department of biostatistics at the Netherlands Cancer Institute<br><br>Data collection IMCISION:<br>Data collection was started from time of consent. Toxicity data were collected from first neoadjuvant ICB infustion until 100 days later. Data were collected in the NKI Head & Neck Surgery and Oncology outpatient clinic, day-care unit and, postoperatively, the NKI Head & Neck Surgery and Oncology ward. Data were entered in an eCRF by the clinical trial department of the NKI. Pathological response was determined by an experienced head and neck pathologist based on the surgical specimen obtained in week 5, or, ultimately, week 6. Radiological response was performed using RECIST (v.1.1) criteria on MR imaging obtained at baseline and week 4. Long term disease status and survival data will be collected for up to 2 years after surgery. The first patient was registered in the trial on February 2nd, 2017 and the final patient on October 25th, 2019. Data cut-off for the present report was May 11, 2021. The clinical eCRF database is securely stored at the NKI, Amsterdam, The Netherlands.
Outcomes	Outcomes NABUCCO trial:<br>The pre-defined primary outcome measure, feasibility (surgery within <12 weeks from start of study treatment), was determined by the PI together with the study biostatistician. Date of treatment start and surgery were verified in the clinical source data (patient files) by trial data managers and inserted in the eCRF. Clinical data was monitored by the study monitor. Duration to surgery was calculated and summarized by the study bioinformatician.<br>The main secondary endpoint, pathological complete response (pCR), was also pre-specified by the PI and biostatistician. Pathological response was determined by an experienced GU pathologist. Only complete absence of neoplastic cells (pT0N0) was marked as pCR. For the purpose of this study response was assessed as treatment repsonse and evaluated by pathological response assessment on radical surgery. Tumor with a complete pathological response (ypT0N0) or residual disease (<=ypT1N0) were classified as responders and tumors with a >=ypT2N0 were classified as non-responders.<br><br>IMCISION outcomes:<br>Primary objectives for the phase Ib (n=12) were feasibility and safety. All patients were monitored for adverse events (AEs) from start of ICB until 100 days after the last ICB treatment. Adverse events were scored according to the Common Terminology Criteria for Adverse Events (CTCAE) version 4.0.3. Feasibility was determined based on immune-related adverse events leading to delay in surgery past week 6. Two neoadjuvant regimens [nivolumab monotherapy (n = first 6 patients) and nivolumab + ipilimumab (n = next 6 patients)] were tested in phase Ib. Safety interim analyses were performed after inclusion of 3 patients in both treatment cohorts. Neoadjuvant ICB would be deemed unsafe and unfeasible if immune-related AEs led to delay in surgery beyond week 6 in more than 1 of 6 patients included in a treatment cohort.<br>Once safety had been established, accrual was expanded into a phase IIa cohort of 20 patients, for which the primary outcome was pathological efficacy and its relation with MRI-based RECIST radiological response. Pathological response was established by determining the percentage of residual viable tumor cells in the surgically resected specimen in week 5 (ultimately week 6). To correct for a low viable tumor cell count already present at baseline (i.e. not a treatment effect), the percentage change in the percentage of viable tumor cell count was calculated from baseline biopsy to week 5–6 surgical specimen. Radiological efficacy was defined according to RECIST (v.1.1) criteria applied on MR imaging performed at baseline and week 4–5 (shortly prior to surgery).<br><br>Secondary and translational outcome measures included 2-year toxicity and survival parameters, baseline and on-treatment tumor hypoxia and molecular and immunological correlates of response to ICB. These were respectively assessed by evaluating trial patients at regular outpatient clinic visits, and by performing correlative DNA/RNAsequencing, multiplex immunofluorescence and IHC studies of patient samples at both time points.<br><br>For this manuscript response from IMCISION was assessed as response to treatment, which was evaluated by pathological response assessment on surgery and by surgery and a decrease of 90-100% in tumor cells from baseline to on-treatment were classified as major pathological responders (MPR), tumors with <=50% TCP at surgery and a decrease of 50-89% in tumor cells from baseline to on-treatment were classified as partial pathological responders (PPR); else tumors were classified as no pathological responders (NPR). Patients with a MPR were classified as responders, and patients with a PPR or NPR were classified as non-responders.
