# Supplementary material for: Spatial relationships in the urothelial and head and neck tumor microenvironment predict response to combination immune checkpoint inhibitors
Source: Nat Commun. 2024 Mar 21;15:2538. doi: 10.1038/s41467-024-46450-1 (PMC10957922; doi:10.1038/s41467-024-46450-1)
Supplement: Supplementary file 2 — Peer Review File [file 41467_2024_46450_MOESM2_ESM.pdf]

nature portfolio

Peer Review File

Spatial relationships in the urothelial and head and neck tumor microenvironment predict response to combination immune checkpoint inhibitors

Open Access This file is licensed under a Creative Commons Attribution 4.0 International License, which permits use, sharing, adaptation, distribution and reproduction in any medium or format, as long as you give appropriate credit to the original author(s) and the source, provide a link to the Creative Commons license, and indicate if changes were made. In the cases where the authors are anonymous, such as is the case for the reports of anonymous peer reviewers, author attribution should be to 'Anonymous Referee' followed by a clear attribution to the source work. The images or other third party material in this file are included in the article's Creative Commons license, unless indicated otherwise in a credit line to the material. If material is not included in the article's Creative Commons license and your intended use is not permitted by statutory regulation or exceeds the permitted use, you will need to obtain permission directly from the copyright holder. To view a copy of this license, visit http://creativecommons.org/licenses/by/4.0/.

REVIEWER COMMENTS

Reviewer #1 (Remarks to the Author):

The authors present a well described and executed study in which they explore how some spatially derived metrics between tumor, non-tumor, and immune components of the tumor microenvironment could be leveraged to predict response to ICI in UC based on pre-treatment specimens.

Although some of the reasoning behind why only examining 1-NN statistics in these data were not entirely clear to me, the approach of applying parametric modeling of the distribution of these spatial metrics is interesting and the comparison to more conventional cell-type density metrics was well done and insightful.

The inclusion of an independent validation cohort from a different anatomic site with rather different tumor biology proved valuable. The findings and models from the UC cohort showed clear validation in the HNSCC (largely HPV-) cohort, both in terms of simple summary statistics like median 1-NN distances and the parametric modelling of the distribution of 1-NN distances. As has generally been the case with biomarkers that are designed to predict ICI response, these tend to be similar across tumor types - perhaps because of the inclusion of features extrinsic to the specific tumor but rather the response of the host immune system and microenvironment.

Reviewer #2 (Remarks to the Author):

This study of mIF to identify immunohistochemical signatures that can be utilized to identify responders to immunotherapy utilizes a novel computational method of assessing the TME, by assessing spatial relationships compared to some of the other published quantitative and qualitative methods that have been published.

Determining biomarkers for response to immunotherapy is important as the utilization of IO for cancers has increased, but may be presented as an alternative treatment to other conventional therapies such as chemotherapy. Thus, predictors of response are important

to optimize therapy.

The study describes their modeling technique and identifies signatures that can be used to predict response to IO in UC, and subsequently validates this in a head and neck cancer cohort. The sample sizes are small, so future studies with more patients will be needed, but it appears to be a promising technique that warrants future study/validation. The authors should consider providing slightly more detail about the clinical characteristics of the cohorts used, including where tissue was obtained from and brief details of treatment characteritics of the cohort (e.g., some may not be familiar with NABUCCO trial).

Reviewer #3 (Remarks to the Author):

Gil-Jimenez et al. performed a study where they mathematically assessed tumor microenvironment (TME) immune cell densities, exclusion ratios, and spatial relationships (SRs) of various cell types using multiplex immunofluorescence data of 24 urothelial cancer (UC) samples treated pre-operatively with combined immune checkpoint inhibitors (ICIs), namely ipilimumab (anti-CTLA-4) and nivolumab (anti-PD-1). By approximating the First-Nearest neighbor (1-NN) distances of cell-cell pairs with a Weibull distribution, the authors found that SRs were a more predictive measure for combined ICI response than traditional density and exclusion ratio metrics. Specifically, the authors showed that higher proximity of CD8+ T-cells to cancer cells or macrophages to cancer cells correlated significantly with positive response to combined ICI in UC. To test the generalizability of their method, the authors applied their SR model to 25 head and neck squamous cell carcinoma (HNSCC) samples similarly treated with combined ipilimumab and nivolumab and showed that it could predict response in this cancer type as well. Through additional analyses, the authors also demonstrated that using the conventional G-function to model 1-NN distributions can be unreliable due to its dependence on specific thresholds, and instead called for the threshold-independent Weibull distribution as a superior approach for quantifying SRs and predicting combined ICI response.

Comments

1. The term “pre-treatment” in the third sentence of the abstract is misleading and can imply pre-treatment with ICIs. Samples used in this study were tumors that had undergone neoadjuvant ICI treatment. “Pre-operative” would be a better choice here.

2. The concluding statement of the abstract, specifically “SRs, in contrast to density metrics, are strong biomarkers of response to ICIs” needs to be reconsidered. This study found that SR metrics correlate with combined ICI treatment response, not ICI treatment in general.

3. The Weibull parameters “scale” and “shape” are inadequately defined. One could interpret “scale” as indicative of a distance metric as seen in Figure 2C where a low scale results in closer proximity between black and pink cells (and vice versa). However, this leaves “shape” up for interpretation. A better explanation of these two terms, especially “shape,” is needed to understand what is being presented in Figure 2 (and subsequent figures thereof) more fully.

4. What threshold, if any, is being used to define “short distances” (line 129) and closeness of two cells? Further, how close must two cells be for biologically meaningful interaction?

5. As the authors note in line 138, SRs can vary depending on the perspective of reciprocal cell-cell pairs (i.e., cell type A to B vs. B to A). However, there is no explanation as to why such a discrepancy exists. Also, scale and shape parameter analyses for UC samples (Supplementary Figure 4) were performed for “Cancer cell to CD8 T-cell” interactions whereas the same analyses were done for “CD8 T-cell to Cancer cell” in HNSCC samples (Supplementary Figure 8A and 8B). It is hard to justify why there is this inconsistency.

6. It is unclear what figure lines 213-217 are referring to (initial read suggests Supplementary Figure 3 but this was not the case). Moreover, the descriptive caption of Supplementary Figure 3A erroneously states “response groups (x-axis)” when the figure shows Fold change (log2) as the x-axis.

7. There are significant outliers in the boxplots for “CD8 T-cell to Negative cell” and “Macrophages to Negative cell” SRs in Figure 4E that may be skewing the median upward

and therefore affecting the significance between responders vs. non-responders. Have the authors considered excluding these outliers and seeing if it still leads to statistical significance?

8. From the discussion (lines 333-341) and data (Figure 4), it is as-yet difficult to see how exclusion ratios and densities have no bearing on SR distance metrics. As the authors note, tumors with an immune excluded phenotype show increased CD8 T-cells in the stroma due to mechanisms that prevent T-cells from reaching the tumor, and the authors call for therapeutic strategies that permit CD8 T-cells to migrate closer to tumor cells. A more responsive tumor, by this definition, would have an increased tumor:stroma T-cell ratio and the resulting distance between T-cells and cancer cells would (by expectation) be lower. An explanation is needed to understand why this expected association is not apparent.

Reviewer #1

Reviewer comments:

The authors present a well described and executed study in which they explore how some spatially derived metrics between tumor, non-tumor, and immune components of the tumor microenvironment could be leveraged to predict response to ICI in UC based on pre-treatment specimens.

Although some of the reasoning behind why only examining 1-NN statistics in these data were not entirely clear to me, the approach of applying parametric modeling of the distribution of these spatial metrics is interesting and the comparison to more conventional cell-type density metrics was well done and insightful.

The inclusion of an independent validation cohort from a different anatomic site with rather different tumor biology proved valuable. The findings and models from the UC cohort showed clear validation in the HNSCC (largely HPV-) cohort, both in terms of simple summary statistics like median 1-NN distances and the parametric modeling of the distribution of 1-NN distances. As has generally been the case with biomarkers that are designed to predict ICI response, these tend to be similar across tumor types - perhaps because of the inclusion of features extrinsic to the specific tumor but rather the response of the host immune system and microenvironment.

Author Response:
First, we thank **Reviewer #1** for the time and effort to assess our manuscript thoroughly and the positive comments on our work regarding the modeling strategy and robustness of the signal found in both the urothelial and head and neck cancer cohorts.

We agree that we could provide a more extensive motivation for selecting the 1-NN distance as a metric in our approach. Our most important motivation was quantifying spatial relationships using a simple and easily interpretable metric: the first nearest-neighbor statistic. Second, since the spatial distances between cell types are an important factor that modulates the interactions between various cell types, we opted for a *distance-based* metric rather than a proportion-based metric, which measures the ratio of two cell types within a given region (such as the K-function[1]).

Changes to the manuscript:
We have adjusted the Introduction (adding reference **[20]**) and Discussion to provide a more elaborate motivation for selecting the 1-NN statistic as our main metric.

References
[1] Parra ER (2021) Methods to Determine and Analyze the Cellular Spatial Distribution Extracted From Multiplex Immunofluorescence Data to Understand the Tumor Microenvironment. Front. Mol. Biosci. 8:668340. doi: 10.3389/fmolb.2021.668340

Reviewer #2

Reviewer comments:

This study of mIF to identify immunohistochemical signatures that can be utilized to identify responders to immunotherapy utilizes a novel computational method of assessing the TME, by assessing spatial relationships compared to some of the other published quantitative and qualitative methods that have been published.

Determining biomarkers for response to immunotherapy is important as the utilization of IO for cancers has increased, but may be presented as an alternative treatment to other conventional therapies such as chemotherapy. Thus, predictors of response are important to optimize therapy.

The study describes their modeling technique and identifies signatures that can be used to predict response to IO in UC, and subsequently validates this in a head and neck cancer cohort. **The sample sizes are small, so future studies with more patients will be needed, but it appears to be a promising technique that warrants future study/validation. The authors should consider providing slightly more detail about the clinical characteristics of the cohorts used, including where tissue was obtained from and brief details of treatment characteristics of the cohort (e.g., some may not be familiar with NABUCCO trial).**

Author Response:
First, we thank **Reviewer #2** for the time and effort invested in assessing our manuscript. We are pleased that the reviewer considers our technique promising and interesting enough for further study and validation.

In response to the reviewer's comment on cohort characteristics, we agree that a summary of clinical trial specifics will be useful for the readers. We have included **Table 1,** summarizing the baseline cohort and tissue characteristics used in this study. Both datasets used in this study were proof-of-concept clinical trials of pre-operative combination ICIs with available multiplex immunofluorescence data. Therefore, the relatively small sample sizes used in this study represent the clinical trial reality of the treatment settings. We agree that future studies will be valuable in further validating our findings.

Changes to the manuscript:
We have included **Table 1,** summarizing the baseline cohort and tissue characteristics used in this study.

Table 1. Clinical trial, treatment, and sample characteristics for the cohorts used in this study.

	**NABUCCO (n=24)** ***NCT03387761***	**IMCISION, Arm B (n=25)** ***NCT03003637***
**Sample size**	24	25
**Cancer type**	Urothelial cancer	Head and neck cancer
**Tissue source**	Bladder, n=24 (100%)	Oral cavity, n=24 (96%)<br>Oropharynx, n=1 (4%)
**Tissue sampling**	Transurethral resection (primary tumor, FFPE)	Primary tumor biopsy (FFPE)
**Treatment type**	Neoadjuvant (pre-operative)	Neoadjuvant (pre-operative)
**Treatment dosage per cycle**	1: ipilimumab<br>2: ipilimumab + nivolumab<br>3: nivolumab	1: ipilimumab + nivolumab<br>2: nivolumab
**Tumor type**	Primary, n=24 (100%)	Primary, n=20 (80%)<br>Recurrence, n=5 (20%)
**HPV-positivity (%)**	n/d	23 (92%)
**Response definition**	Pathological response assessment (complete pathological response or residual disease)	Pathological response assessment combined with comparison of tumor cells decrease from baseline vs. on-treatment sample
**Clinical response (%)**	14 (58%)	9 (36%)
**Clinical Stage**	cT3-4aN0M0 n=14 (58%)<br>cT2-4aN1-3M0 n=10 (42%)	cT2N0 n=5 (20%)<br>cT3-4aN0M0 n=10 (40%)<br>cT2-4aN1-3M0 n=10 (40%)
**Sex (%)**	Male, n=18 (75%)	Male, n=20 (80%)

Abbreviations: n/d: not determined

Reviewer #3

Reviewer comments:

Gil-Jimenez et al. performed a study where they mathematically assessed tumor microenvironment (TME) immune cell densities, exclusion ratios, and spatial relationships (SRs) of various cell types using multiplex immunofluorescence data of 24 urothelial cancer (UC) samples treated pre-operatively with combined immune checkpoint inhibitors (ICIs), namely ipilimumab (anti-CTLA-4) and nivolumab (anti-PD-1). By approximating the First-Nearest neighbor (1-NN) distances of cell-cell pairs with a Weibull distribution, the authors found that SRs were a more predictive measure for combined ICI response than traditional density and exclusion ratio metrics. Specifically, the authors showed that higher proximity of CD8+ T-cells to cancer cells or macrophages to cancer cells correlated significantly with positive response to combined ICI in UC. To test the generalizability of their method, the authors applied their SR model to 25 head and neck squamous cell carcinoma (HNSCC) samples similarly treated with combined ipilimumab and nivolumab and showed that it could predict response in this cancer type as well. Through additional analyses, the authors also demonstrated that using the conventional G-function to model 1-NN distributions can be unreliable due to its dependence on specific thresholds, and instead called for the threshold-independent Weibull distribution as a superior approach for quantifying SRs and predicting combined ICI response.

Author Response (including some comments from Reviewer #3)**:**

We thank **Reviewer #3** for the time and effort to assess our manuscript and the constructive suggestions provided.

Comments

1. The term “pre-treatment” in the third sentence of the abstract is misleading and can imply pre-treatment with ICIs. Samples used in this study were tumors that had undergone neoadjuvant ICI treatment. “Pre-operative” would be a better choice here.

Author response:
We see how the term could be ambiguous. We performed the translational analysis on transurethral resections collected at diagnosis (before the combination ICI treatment started).

Changes to the manuscript:
We have reworded the sentence, as suggested by the reviewer: “*We quantified the TME immune cell densities and spatial relationships (SRs) using the multiplex immunofluorescence data of 24 baseline UC samples, obtained before pre-operative combination ICI treatment”.*

2. The concluding statement of the abstract, specifically "SRs, in contrast to density metrics, are strong biomarkers of response to ICIs" needs to be reconsidered. This study found that SR metrics correlate with combined ICI treatment response, not ICI treatment in general.

Author response:
We agree with the reviewer that we should have phrased this more carefully, as we only intended to make statements about this study's data and analyses.

Changes to the manuscript:
We have adjusted the abstract and conclusions to reference "pre-operative combination ICIs" to emphasize that the biomarkers we obtained are associated with response to *combination* immune checkpoint inhibitors treatment.

3. The Weibull parameters "scale" and "shape" are inadequately defined. **One could interpret "scale" as indicative of a distance metric**, as seen in Figure 2C where a low scale results in closer proximity between black and pink cells (and vice versa). **However, this leaves "shape" up for interpretation. A better explanation of these two terms, especially "shape," is needed to understand what is being presented in Figure 2** (and subsequent figures thereof) more fully.

Author response:
As per the definition of the Weibull function, the scale parameter is a numerical parameter that increases with distributions that exhibit increasing **spread,** are wider. In **Reviewer Figure 0A** we fixed the shape parameter to **1.5** and varied the scale parameter from 25 to 230. The resulting density functions are shown in **Reviewer Figure 0B,** demonstrating how the spread of the Weibull function increases with scale.

The shape parameter, on the other hand, is a parameter that is **not directly** related to the position of the **"mean"** or **"spread"** of the distribution. It is a parameter that, when its values change, gives rise to qualitatively different forms (or "shapes", if you will) of the density function. For example, when shape < 1, the distribution resembles a semi-exponential distribution (**Reviewer Figure 1A**). However, when shape > 1, the distribution can resemble a right-skewed distribution (**Reviewer Figure 1B**), a Gaussian-like distribution (**Reviewer Figure 1C**), or a left-skewed distribution (**Reviewer Figure 1D**) - which are very different distributions in nature.

In our analysis, we allowed the parametric modeling to adopt any positive shape value. Nevertheless, as seen in **Reviewer Figure 0A,** the value of the shape parameter (mean value of ~2) matched with a non-exponential distribution (shape > 1) in almost all cases. The few cases (see red ellipse in **Reviewer Figure 0A**) in which the shape parameter matched with a semi-exponential distribution (shape < 1) can be attributed to noisy distributions, as it is biologically impossible to have such behavior in the first-nearest neighbor distribution.

Within the range of values of shapes that the model learned, it can be seen in **Reviewer Figure 0** that an increase of the shape parameter for fixed high values of scale (panels **C/D**) and fixed low values of scale (panels **E/F**) (horizontal arrows) allowed the distribution to accommodate distributions with distinct behaviors of right-skewed distributions that altogether match with different scenarios of summarized spatial relationships.

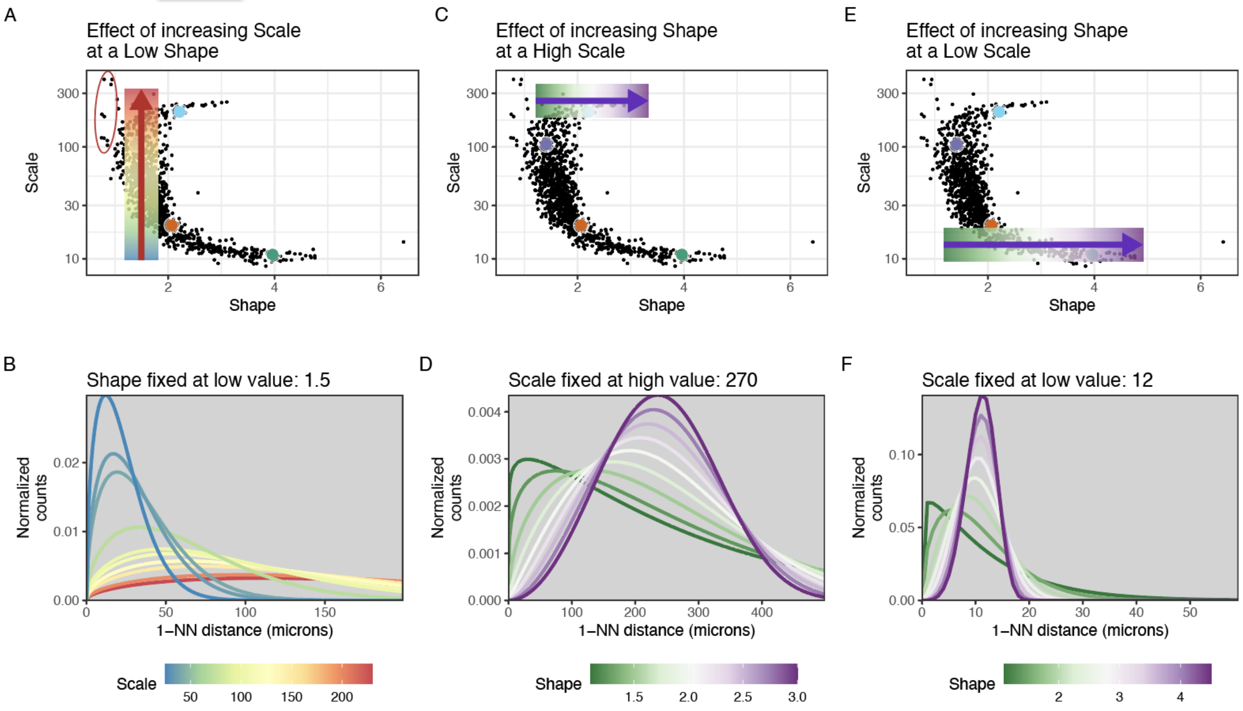

Reviewer Figure 0. Effect of the modeled shape and scale parameters on the Weibull distribution.
A-B) Differences at the Weibull distribution when increasing the scale parameter at a fixed shape.
C-D) Differences at the Weibull distribution when increasing the shape parameter at a fixed scale (high scale).
E-F) Differences at the Weibull distribution when increasing the shape parameter at a fixed scale (low scale).

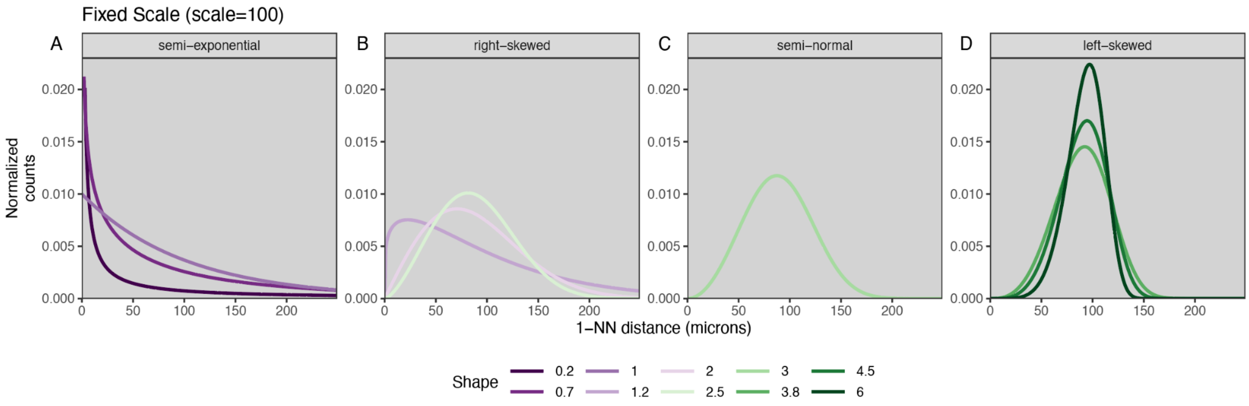

Reviewer Figure 1. Representation of a Weibull distribution when altering the shape parameter at a fixed scale. Each facet groups Weibull distributions with a similar behavior.

The mean and median of the Weibull distribution can be analytically estimated from the shape and scale parameters (**Eqs. R1-R2**) using a non-linear function. From such equations, it can be seen that neither shape nor scale directly relates to the mean or the median, as they clearly show a non-linear relationship.

$$Median_{1NN\ distance} = scale\ (ln2)^{1/shape} \quad (Eq\ R1)$$
$$Mean_{1NN\ distance} = scale\ \Gamma(1 + 1/shape) \quad (Eq\ R2)$$

We recognize that interpreting such abstract parameters is difficult, and this motivated us to show, on top of the associations of scale and shape vs. clinical response, the associated differences based on median 1-NN distances vs. clinical response, as depicted in **Figure 4E**.

Changes to the manuscript:
We have changed the Results section to provide more clarity on the parameter interpretation. Moreover, to complement our explanations we have included **Reviewer Figures 0 and 1** in the manuscript as **Supplementary Figure 2** and **Supplementary Figure 3**.

5. As the authors note in line 138, SRs can vary depending on the perspective of reciprocal cell-cell pairs (i.e., cell type A to B vs. B to A). **However, there is no explanation as to why such a discrepancy exists**.

Author response:
We attempted to explain this lack of symmetry in the manuscript by stating that "*the statistic is measured between a reference cell type (cell from) and a target cell type (cell to). The distance between each reference cell type and their closest target cell type yielded a 1-NN vector*". Consequently, the 1-NN distance vector depends on the reference and target cell type choice. We illustrate the lack of symmetry behavior in **Reviewer Figure 2**, where the left shows how to calculate the 1-NN distances as it was originally depicted in the manuscript (**Figure 1E,** 1-NN from a T-cell to the nearest cancer cell), but on the right, we now show what the 1-NN looks like if we swap the reference vs. target cell types. If we do so and study the statistic from cancer cells to the nearest T-cell, the statistic is measured from each cancer cell, yielding a vector of 5 distances (*right panel, thick, dotted straight lines*). Even in such a simple example, the perspective matters when studying a spatial relationship, and such relationships are, by definition, not symmetrical except for same-cell relations (e.g., cancer cell to cancer cell, B-cell to B-cell, …). For interpretability purposes, the median 1-NN distances are also highlighted in the plots (thick dotted red lines), showing that changing the SR perspective also changes the median 1-NN distances.

Editorial Note: Icons from panel were adapted from bioIcons (cancerous-cell-1, lymphocytes-4, t-lymphocyte licensed under CC-BY 3.0 Unported by Servier).

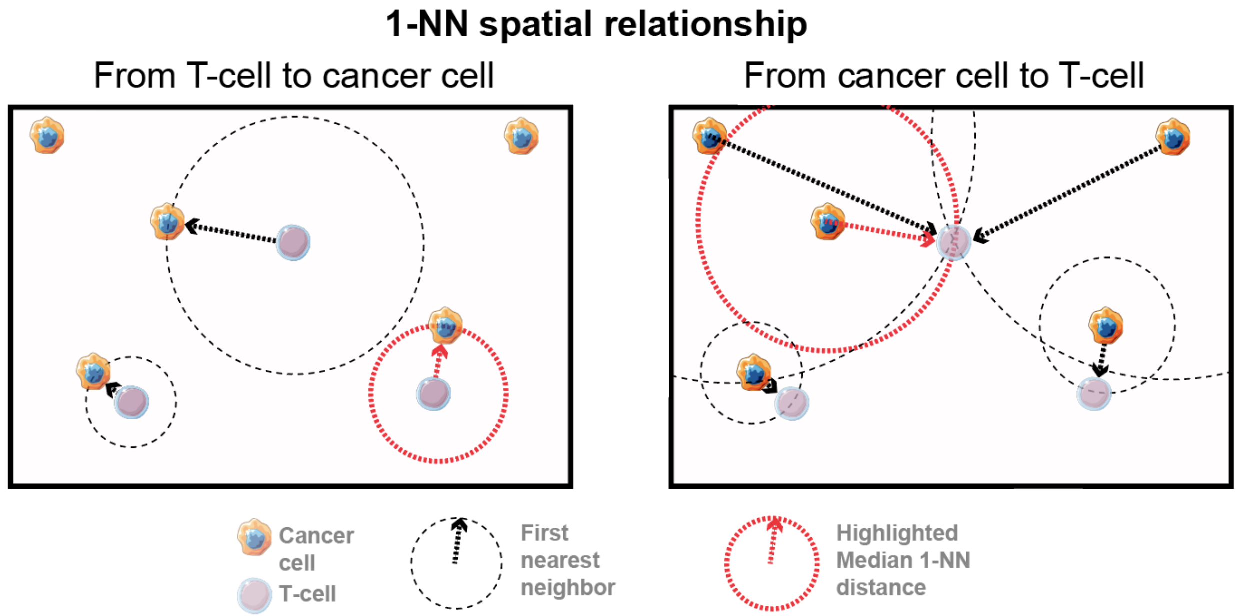

Reviewer Figure 2. SRs were summarized using the 1-NN statistic studied from a reference cell type to a target cell type. The 1-NN statistic is not symmetrical; panels A and B illustrate the statistic when T-cells (left panel) or cancer cells (right panel) are selected as a reference cell type, respectively. Only the 1-NN distances are drawn in each panel. Distances in red indicate the median 1-NN distance for that particular SR.
Abbreviations: *SR*: spatial relationship.

Changes to the manuscript:
We have added this illustration in **Supplementary Figure 4** to the manuscript.

Also, scale and shape parameter analyses for UC samples (Supplementary Figure 4) were performed for "Cancer cell to CD8 T-cell" interactions. In contrast, the same analyses were done for "CD8 T-cell to Cancer cell" in HNSCC samples (Supplementary Figure 8A and 8B). It is hard to justify why there is this inconsistency.

Author response:
We thank the reviewer for picking this up. We made a typo in **lines 235-239:** "Supplementary Figure **3**A-E" should have been read "**Supplementary Figure 4A-E**". We see how this could have created confusion regarding the interpretation of our findings: "*We identified an association between the SR from cancer cells to CD8+ T-cells and response with a small fold change for the shape parameter ($|FC_{shape}|$=0.11, FDR_{shape}=0.09), pointing to a difference in distribution that was not detected in terms of median 1-NN distances (**Supplementary Figures 4A-E**, p=0.98), suggesting that this may well be a false positive*". As noted in the text, the validation approach in the head and neck cancer cohort was only done for the strongest biomarkers hits (defined as FDR<0.04 from **Figures 4C-D**), which were "*CD8 T-cell to cancer cell*" and for "*Macrophage to cancer cell*".

Changes to the manuscript:

We corrected the typo to refer to the correct Supplementary figure and we adjusted the title from **Supplementary Figure 4** to "*Association of spatial relationships with response to pre-operative ipilimumab+nivolumab in urothelial cancer in hits with a weak association*". We also re-labeled a typo in the y-axis from **Supplementary Figure 4D** as it should have been labeled as 'Shape'. Since we added three supplementary figures to the revised manuscript, we needed to update the numbering and the 'old' reference to **Supplementary Figure 4** is now **Supplementary Figure 7**.

6. It is unclear what figure lines 213-217 are referring to (initial read suggests Supplementary Figure 3 but this was not the case). Moreover, the descriptive caption of Supplementary Figure 3A erroneously states "response groups (x-axis)" when the figure shows Fold change (log2) as the x-axis.

Author response:
Lines 213-217 refer to three hits representing the association between SRs quantified by the G-AUC-T approach and response to treatment when using thresholds of T larger than 25 microns. As they were not significantly associated with clinical response when assessed at a 25-micron threshold (G-AUC-25), they were not labeled in **Supplementary Figure 3A** as only significant hits are labeled in volcano plots in the manuscript.

Changes to the manuscript:
To avoid confusion, we have added a volcano plot on the associations with the G-AUC-50 vs. response (G-function evaluated at 50 microns) and labeled the significant hits in **Supplementary Figure 3C** (used to be Supplementary Figure 3B) and adjusted the text to make the reference to the figure clear. Lastly, we have also adjusted the figure caption from **Supplementary Figure 3A** to state that we evaluated the $\log_2$ fold change between response groups.
Note, **Supplementary Figure 3** is now **Supplementary Figure 6**.

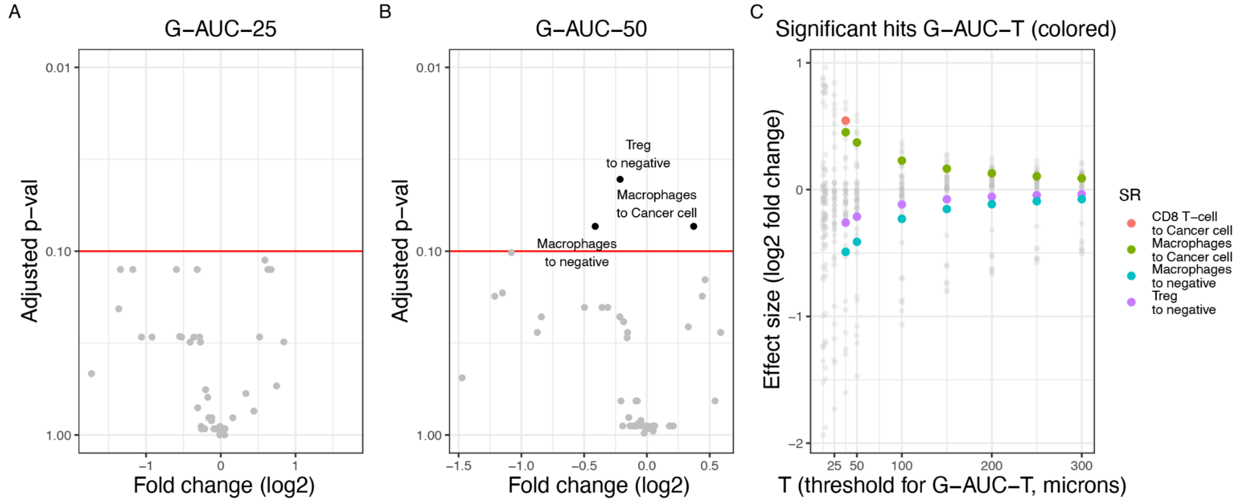

Supplementary Figure 6. **Association of spatial relationships derived from the G-function with response to pre-operative ipilimumab+nivolumab in urothelial cancer.**

(A) Volcano plot showing the fold change on the G-function parameter evaluated at 25 microns (G-AUC-25) between response groups (x-axis, log_2 fold change) and statistical significance by Wilcoxon test adjusted by multiple hypothesis testing (y-axis).
(B) Volcano plot showing the fold change on the G-function parameter evaluated at 50 microns (G-AUC-50) between response groups (x-axis, log_2 fold change) and statistical significance by Wilcoxon test adjusted by multiple hypothesis testing (y-axis).
(C) Fold change (y-axis, log_2 scale) on the G-function parameter evaluated at different thresholds T (x-axis) between response groups. Fold changes significantly associated with response as evaluated with a Wilcoxon test adjusted by multiple hypothesis testing are depicted in color. Non-significant fold changes are depicted in grey..
Unless otherwise stated, all statistical tests were two-sided.
Abbreviations: *G-AUC-T*: G-function evaluated at a threshold T; *T*: threshold; *AUC*: Area under the curve.

7. There are significant outliers in the boxplots for "CD8 T-cell to Negative cell" and "Macrophages to Negative cell" SRs in Figure 4E that may be skewing the median upward and therefore affecting the significance between responders vs. non-responders. Have the authors considered excluding these outliers and seeing if it still leads to statistical significance?

Author response:
We thank the reviewer for this insightful comment. For interpretation purposes, within the candidate list of spatial relationships significantly associated with response, we reported differences between the median 1-NN distances of the response groups (**Figure 4E**). Because the median distances were not the direct output from our parametric modeling (non-linear mixed effect model), we tested for such differences using a rank test (2-sided Mann-Whitney test). As rank-based tests are not affected by outliers in the same way parametric tests are, extreme values do not excessively influence the outcome. Due to this robustness, the outlier shown in the boxplot does not affect the reported non-parametric test results. Moreover, since the data point behaved *normally* for the scale and shape parameter, we do not believe the observation to be an artifact and have not removed it from our study.

Changes to the manuscript:
We have adjusted the text to clarify the test type used for differences between median 1-NN distances.

8. From the discussion (lines 333-341) and data (Figure 4), **it is as-yet difficult to see how exclusion ratios and densities have no bearing on SR distance metrics.** As the authors note, tumors with an immune excluded phenotype show increased CD8 T-cells in the stroma due to mechanisms that prevent T-cells from reaching the tumor, and the authors call for therapeutic strategies that permit CD8 T-cells to migrate closer to tumor cells. **A more responsive tumor, by this definition, would have an increased tumor:stroma T-cell ratio and the resulting distance between T-cells and cancer cells would (by expectation) be lower**. An explanation is needed to understand why this expected association is not apparent.

Author response:

We thank the reviewer for raising this point, as it is an important aspect of our findings. We have studied exclusion ratios extensively. As we showed in our appendix, we simulated data to study the relationship between densities, exclusion ratios, and SR metrics. Our analysis reveals situations where the exclusion ratio and SR are associated (**Supplementary Notes Figure 1C-D, Supplementary Notes Figure 2B**). More importantly, the relationship between abundance and spatial relationship metrics depends on the context and perspective of the SR and the relative abundance of the cell types associated with the SRs. It is important to note that the exclusion ratio metric is a simple metric that does not capture the full complexity of the TME, as it is solely based on the *ratio* between intratumoral and stromal density, which both assume a homogeneous cell spatial distribution and do not take variation in spatial distributions into account. Importantly, the SR metrics do not rely on such assumptions.

Following the observations of the potential relationship between spatial and abundance metrics from our simulated data, in our cohort, we tested for 1) association between response and immune cell densities (**Figure 4A**), 2) association between response and exclusion ratios (**Figure 4B**), as well as 3) association between response and SR metrics (**Figure 4C-D**). Such analyses allowed us to conclude that densities and exclusion ratios are not different between response groups, but some SR metrics are. Importantly, the SRs that did associate with response were not associated with exclusion ratios, and we believe the SRs are better capable of capturing the complex distance relations between cells than exclusion ratios. These generalizations are consistent with the absence of predictive power of exclusion ratios when SRs are already included, as shown in **Figure 4E.**

Changes to the manuscript:
We have adjusted the Discussion to clarify this point.

REVIEWERS' COMMENTS

Reviewer #1 (Remarks to the Author):

The authors have addressed my comments.

Reviewer #2 (Remarks to the Author):

The authors took into account the reviewers comments and addressed them with the additions and edits. I do not have additional edits at this time.

Reviewer #3 (Remarks to the Author):

Thank you for addressing the concerns outlined in my initial review. No further comments at this time.
